# A chromosome-level genome assembly provides insights into the environmental adaptability and outbreaks of *Chlorops oryzae*

**DOI:** 10.1038/s42003-022-03850-7

**Published:** 2022-08-26

**Authors:** Ailin Zhou, Cong Huang, Yi Li, Xinwen Li, Zhengbing Zhang, Hualiang He, Wenbing Ding, Jin Xue, Youzhi Li, Lin Qiu

**Affiliations:** 1 Hunan Provincial Key Laboratory for Biology and Control of Plant Diseases and Insect Pests, College of Plant Protection, Hunan Agricultural University, Changsha, 410128 China; 2 Hunan Provincial Engineering & Technology Research Center for Biopesticide and Formulation Processing, Changsha, 410128 China; 3 Shenzhen Branch, Guangdong Laboratory for Lingnan Modern Agriculture, Genome Analysis Laboratory of the Ministry of Agriculture, Agricultural Genomics Institute at Shenzhen, Chinese Academy of Agricultural Sciences, Shenzhen, 518120 China; 4 Plant Protection and Inspection Station, Agriculture and Rural Development of Hunan Province, Changsha, 410005 China

**Keywords:** Entomology, Genome

## Abstract

*Chlorops oryzae* is a pest of rice that has caused severe damage to crops in major rice-growing areas in recent years. We generated a 447.60 Mb high-quality chromosome-level genome with contig and scaffold N50 values of 1.17 Mb and 117.57 Mb, respectively. Hi-C analysis anchored 93.22% scaffolds to 4 chromosomes. The relatively high expression level of *Heat Shock Proteins* (*HSPs*) and antioxidant genes in response to thermal stress suggests these genes may play a role in the environmental adaptability of *C. oryzae*. The identification of multiple pathways that regulate reproductive development (juvenile hormone, 20-hydroxyecdsone, and insulin signaling pathways) provides evidence that these pathways also play an important role in vitellogenesis and thus insect population maintenance. These findings identify possible reasons for the increased frequency of outbreaks of *C. oryzae* in recent years. Our chromosome-level genome assembly may provide a basis for further genetic studies of *C. oryzae*, and promote the development of novel, sustainable strategies to control this pest.

## Abstract (web summary)

A chromosome-level genome assembly for the rice pest, *Chlorops oryzae*, pinpoints molecular pathways that might contribute toward increased outbreaks for this important crop pest.

## Introduction

**Fig. 1:**
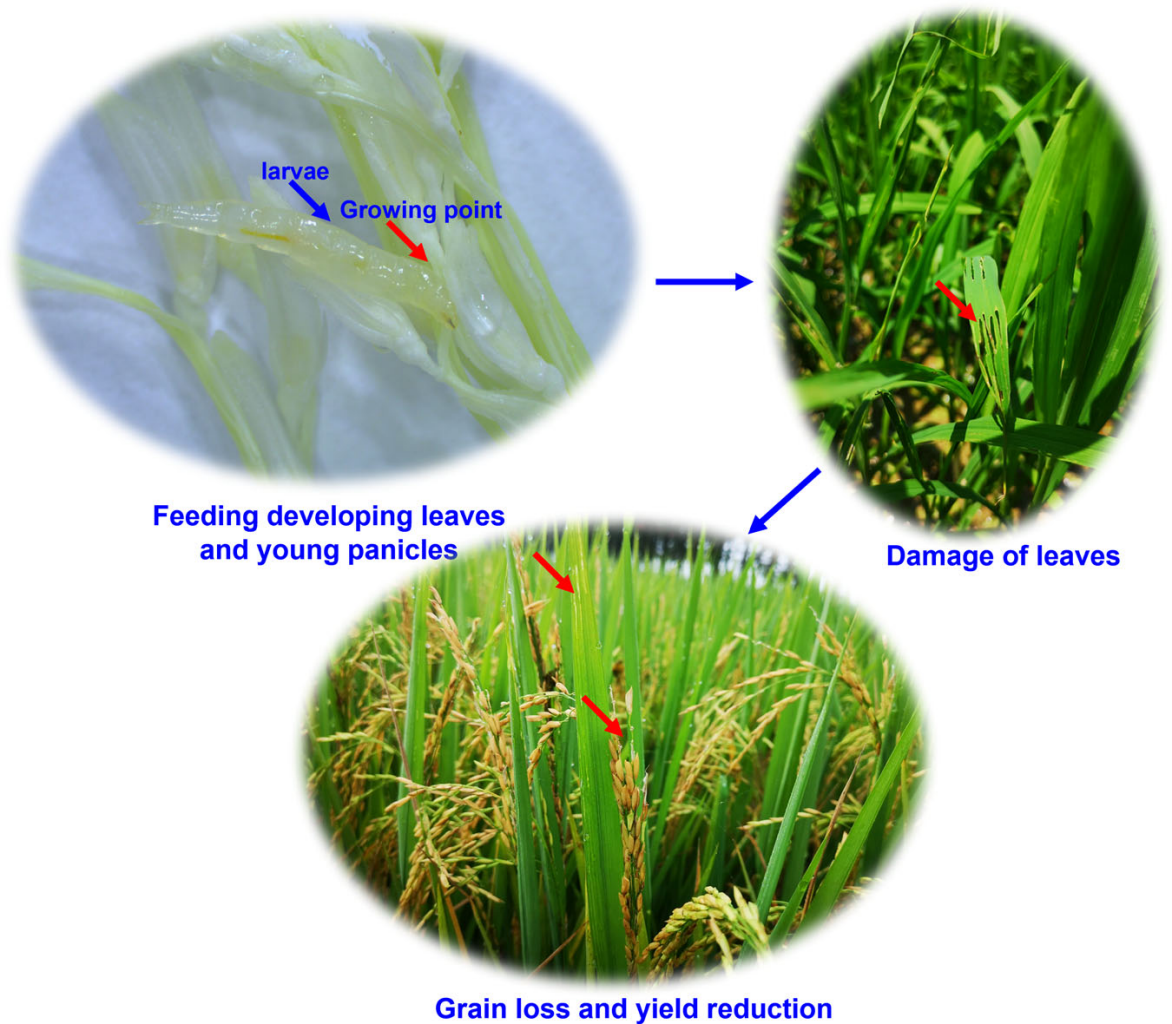
*Chlorops oryzae* larvae damage to rice plants. Red arrow pointing to damage location (growing point, leaves and rice ear).

*Chlorops oryzae* (Diptera, Chloropidae) is an important pest of rice. Newly hatched larvae burrow into the stems of rice plants, then move to the growing tips where they feed on developing leaves and young panicles^1^ (Fig. 1). Over the last century, this species has become widespread throughout Japan and Korea causing severe damage to rice crops^2^. In recent years, it has spread to mountainous and semi-mountainous regions and has caused severe crop damage in the country’s main rice-growing regions, becoming China’s most destructive rice pest. Despite the increasing economic impact of this pest, there is limited data on the major ecological characteristics of *C. oryzae*, such as environmental adaptability and frequency of outbreaks, due to a lack of genomic resources for this organism.

There are ~400 insect species with genomes available at NCBI (https://www.ncbi.nlm.nih.gov/genome/browse#!/overview/insects), and genomic tools, particularly the availability of a high-quality assembled genomes, could help explain the molecular mechanism for *C. oryzae* invasion adaptability and outbreak frequency^3,4^. Several genome sequences have been reported in Diptera taxa, including *Drosophila melanogaster*^5^, *Anopheles gambiae*^6^, *Musca domestica*^7^, *Ceratitis capitata*^8^ and *Bactrocera dorsalis*^4^, which have helped determine the molecular and genetic mechanisms of many biological problems. Unfortunately, no genome, particularly a chromosome-level genome, is available for *C. oryzae*.

Changing environmental conditions such as global warming are known to influence the frequency of insect outbreaks. The growth, development, and reproduction of insects can all be directly affected by temperature. For example, in multivoltine taxa such as the Aphididae and some Lepidoptera (e.g *Pieris brassicae*), higher temperature decreases development time, potentially increasing the number of generations produced per year^9^. Temperature also affects insect distribution and abundance^10^. According to the Intergovernmental Panel on Climate Change (IPCC), the planet is warming by around 0.6 °C per annum, with global temperatures anticipated to rise by an average of 1.4–5.8 °C by 2100^11^. Over the past 55 years, the average number of high-temperature days recorded in China has increased by 28.4%^12^. Consequently, this raises the question of how *C. oryzae* adapts to a warming environment. Furthermore, the emergence of insecticide resistance may be an additional factor influencing pest outbreaks. *C. oryzae* has become widespread and is increasing rapidly, making crop protection more challenging^13^. Insecticides are currently the primary means of controlling *C. oryzae* in the field. Growing evidence suggests that resistance to chemical insecticides is caused by decreased sensitivity of target-site proteins and increased metabolic detoxification of insecticides^14^. Metabolic resistance arises from the overexpression of detoxification enzyme genes, which belong to three major metabolic detoxification gene families: carboxylesterase, glutathione-S-transferases and cytochrome P450^13^. *P450s* are one of the largest gene families in all organisms, performing highly diverse physiological and biochemical functions essential for the detoxification and/or activation of heterologous and endogenous compounds^15^. Understanding the genomic characteristics that underpin high temperature adaptation and insecticide resistance is essential for developing effective prevention and control measures for this pest.

To determine the internal mechanisms underlying the adaptability and frequency of outbreaks of *C. oryzae* in recent years, we generated a high-quality chromosome-level genome assembly of *C. oryzae* through the combined application of PacBio and Illumina sequencing, the HiSeq X Ten platform, and Hi-C. We then conducted a comparative analysis using available insect genomes to gain a better understanding of the genomic evolution of *C. oryzae*. This reference genome has facilitated the identification of xenobiotic detoxification enzymes such as cytochrome P450s. Additionally, by combining transcriptome and qPCR technologies, we further investigated the molecular basis underlying the ability of *C. oryzae* to adapt to novel and changing environmental conditions. Finally, we discuss the results of functional studies on the reproductive development of *C. oryzae*. Our findings may provide a genetic basis for future research on *C. oryzae* outbreaks and promote the development of effective strategies to control this pest.

## Results

### Assembly and annotation of the *C. oryzae* genome

**Fig. 2:**
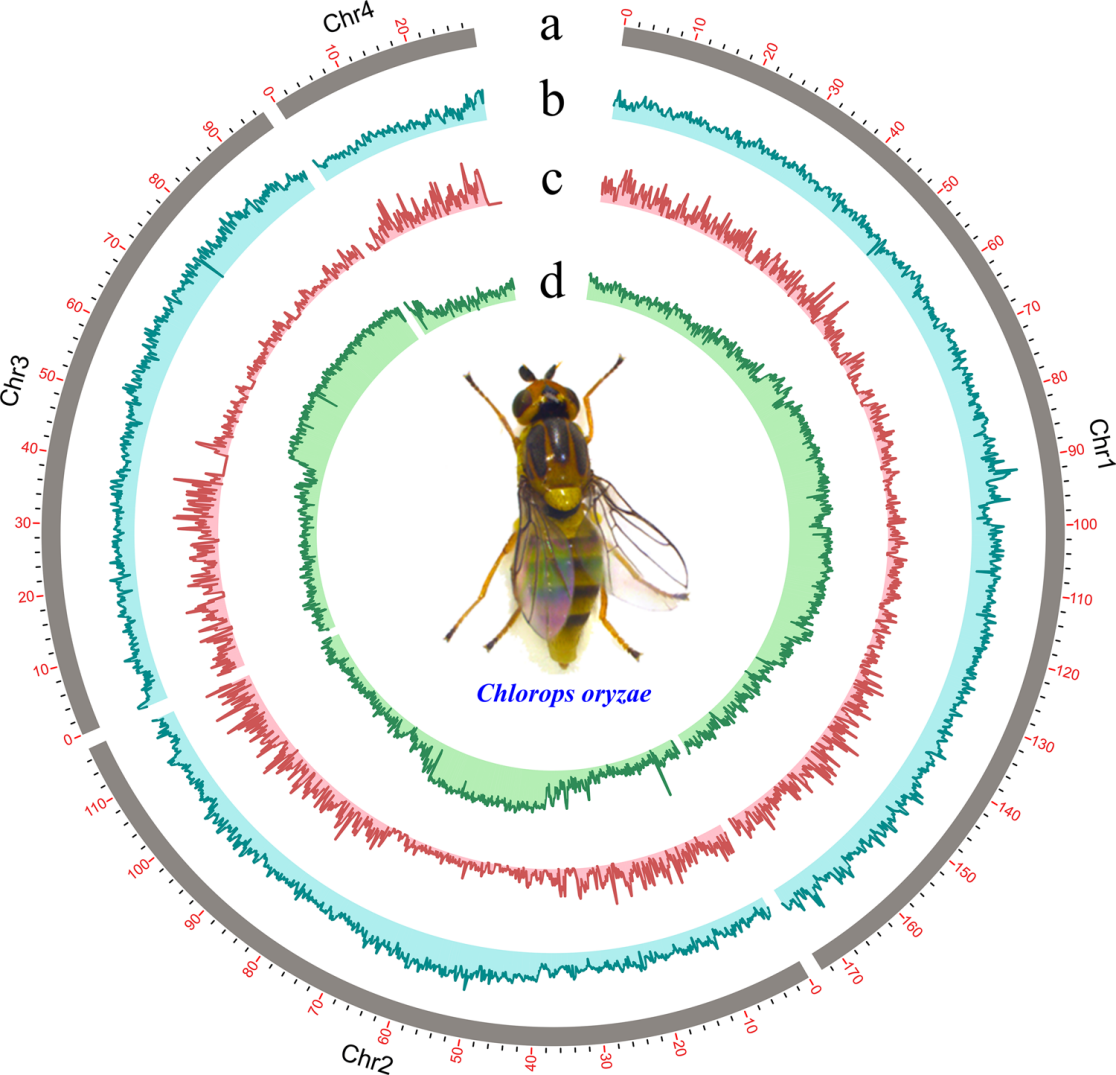
Circular diagram depicting the characteristics of the *Chlorops oryzae* genome. From the outer to the inner circle: **a** 4 chromosome at the Mb scale; **b** GC concent across the genome, drawn in 0.1 Mb non-overlapping windows; **c** gene density across the genome, drawn in 0.1 Mb non-overlapping windows; **d** repeat density across the genome, drawn in 0.1 Mb non-overlapping windows.

*C. oryzae* has a diploid chromosome number (2n) of 8. To analyze this genetic resource, we assembled the *C. oryzae* genome using PacBio long reads and Hi-C chromatin contact information. Removing low-quality and short reads, left a total of 55.92 Gb clean reads for genome assembly, which k-mer analysis estimated to have a genome size of 462.76 Mb (Supplementary Fig. 1 and Supplementary Table 1). A total length of 448.90 Mb assembled genome obtained by using the third-generation sequencing, after correction with Illumina reads, we obtained 3407 contigs with a total length of 447.60 Mb and an N50 of 1.17 Mb. The assembly was then significantly improved, yielding 1575 scaffolds with a total length of 447.78 Mb and an N50 of 117.57 Mb. Hi-C chromatin contact information further supported 4 scaffolds being anchored, ordered, and oriented to give four chromosomes with >93% of assembled bases located on these (Fig. 2, Table 1 and Supplementary Table 2). The completeness of the genome, evaluated by calculating the genome coverage rate for a set of single-copy orthologous eukaryotes genes with BUSCO (v3.0.1), was estimated at 96.1% (Supplementary Table 3). Further analysis of GC content and sequencing coverage showed a normal distribution among assembled scaffolds (Supplementary Fig. 2), which is indicative of low contamination of the assembly. More than 97% of consensus transcripts mapped to the assembly (Supplementary Table 4). These results suggest high accuracy and completeness of the genome assembly.

**Table 1:** Features of the *Chlorops oryzae* genome assembly.

Features	Statistics
Genome size (bp)	447,595,289
Number of chromosomes	4
Number of contigs	3407
Contig N50 (bp)	1,171,122
Number of scaffolds	1575
Scaffold N50 (bp)	117,565,011
GC content (%)	36.09
Repeat (%)	57.38
BUSCO (% complete)	96.1
Percentage of scaffolds in chromosomes (%)	93.22

In total, 256.8 Mb of repeat sequences were identified, comprising 56.30% of the *C. oryzae* genome (Supplementary Table 5). DNA transposons and retroelements accounted for 7.25% and 14.09%, respectively. 5.03% were classified as long interspersed elements (LINEs), 0.01% as short interspersed elements (SINEs) and 9.05% as long terminal repeats (LTRs) of the genome. The protein-coding genes in the reference genome were predicted by EVidenceModeler (EVM) (http://evidencemodeler.github.io/), a total of 17,259 gene models were predicted in the assembled genome as the reference gene set (Supplementary Table 6). Of these genes, 14,863 (86.12%) coding proteins were annotated by functional databases (Supplementary Table 7), including Uniprot, GO, KO, Map, NR, NT, PFAM, and eggNOG. Furthermore, we identified different types of noncoding RNAs (ncRNAs), including 1378 tRNAs with tRNAscan-SE (http://lowelab.ucsc.edu/tRNAscan-SE/), and 161 miRNAs, 130 rRNAs and 93 snRNAs, by referencing known noncoding RNA libraries, Rfam (http://rfam.xfam.org/) (Supplementary Table 8).

### Gene orthology and phylogenetic analysis

**Fig. 3:**
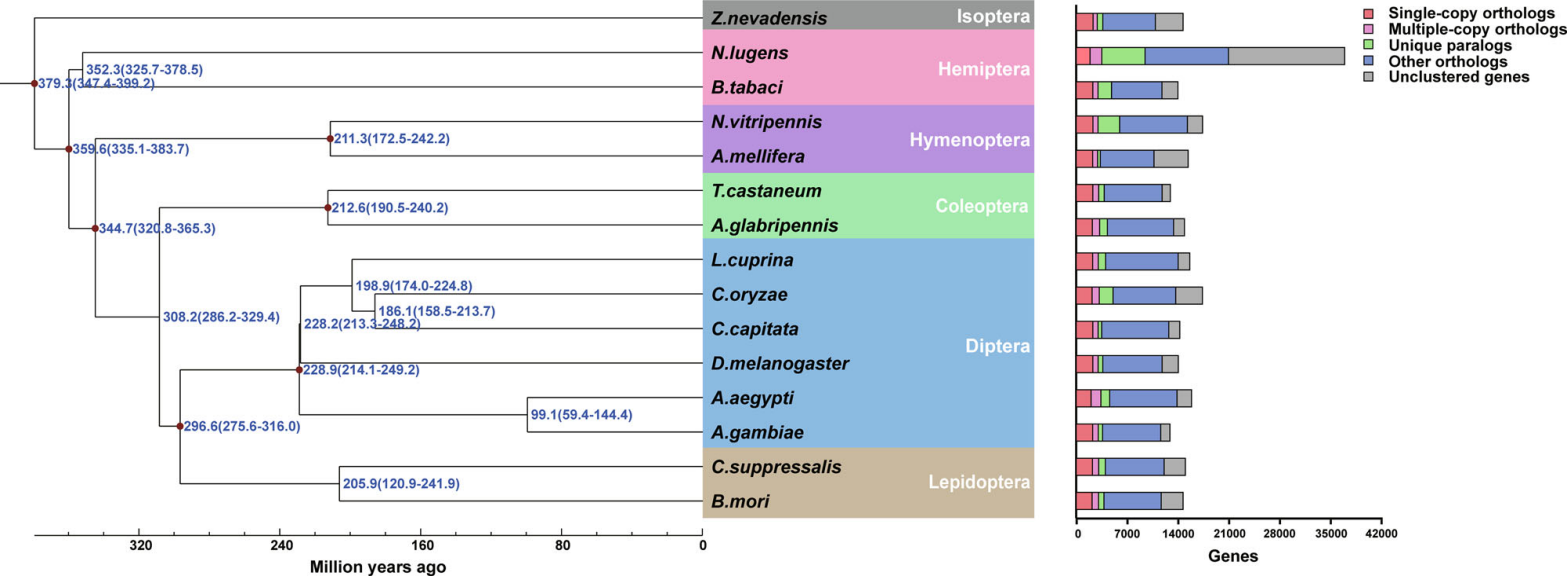
Phylogenetic tree and gene orthology of *Chlorops oryzae* and 14 other insect genomes. Phylogeny inferred from identified single-copy genes in PhyML using the maximum likelihood method (100 bootstrap replicates). Divergences were estimated by the BRMC method using mcmctree.

OrthoMCL (http://OrthoMCL.org/OrthoMCL/) was used to identify orthologous genes in *C. oryzae* and 14 other insect species from six orders (Isoptera, Hemiptera, Hymenoptera, Coleoptera, Diptera and Lepidoptera). A total of 2298 single-copy orthologous genes and 1602 multiple-copy orthologous genes were identified (Supplementary Table 9). Protein sequences of the single-copy genes were used to infer phylogenetic relationships and estimate the divergence between species. The result indicates that *C. oryzae* diverged from *C. capitata* (both of which are members of the Cyclorrhapha) around 186 million years ago (Fig. 3).

### Gene family expansion and contraction

We used CAFÉ software to study the gene family expansion and contraction of *C. oryzae* and related species during evolution. The results showed that compared with the common ancestor of *C. oryzae* and *C. capitata*, the *C. oryzae* genome displayed 561 expanded and 416 contracted gene families (Supplementary Fig. 3).

### The *C. oryzae* cytochrome P450 gene

**Fig. 4:**
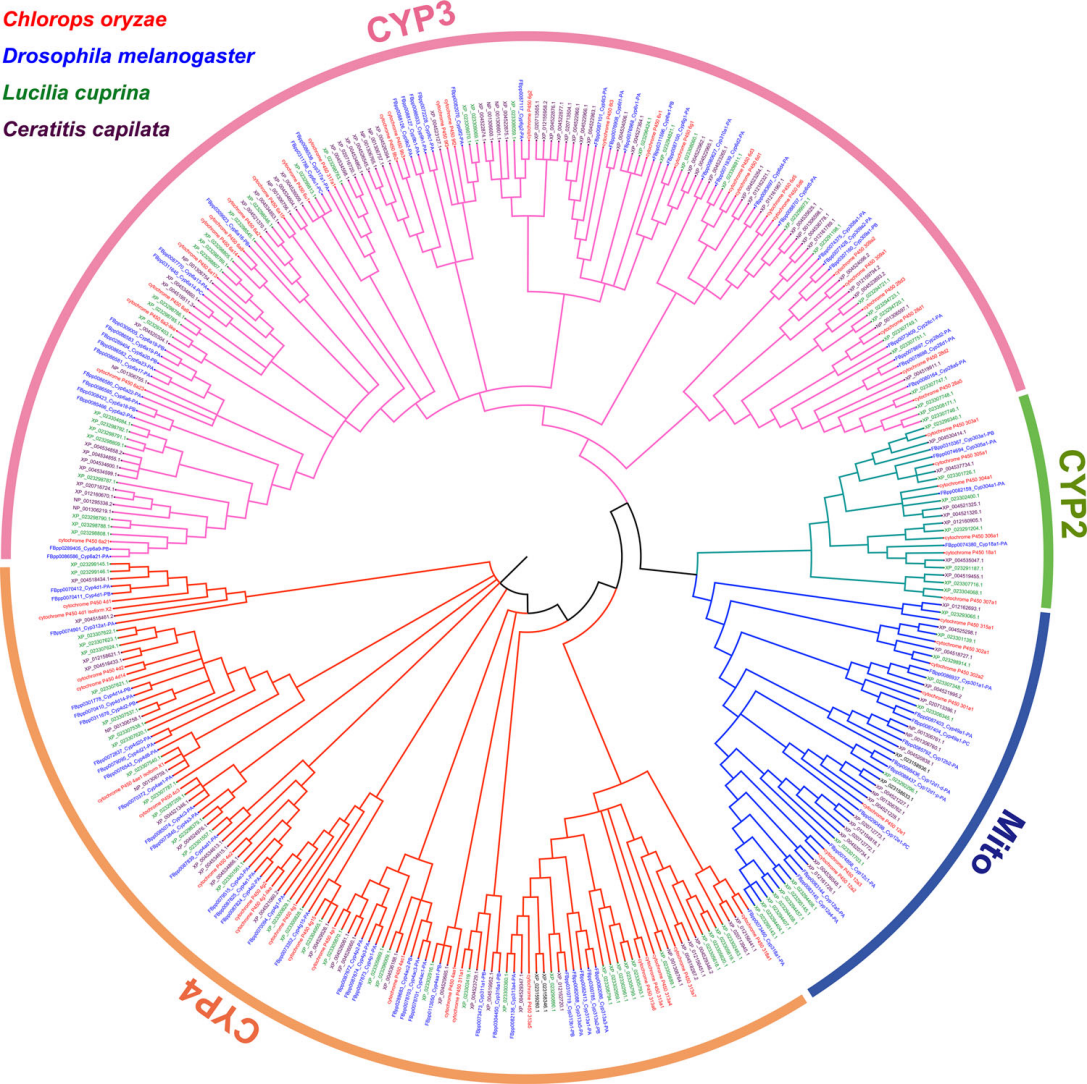
Phylogenetic tree of the cytochrome P450 (P450) gene family of *Chlorops oryzae* and other insects. The phylogenetic tree of the P450 gene family was constructed using genes from *Chlorops oryzae*, *Drosophila melanogaster*, *Lucilia cuprina*, and *Ceratitis capilata*. The Neighbor-Joining (NJ) tree was constructed using MEGA v.7.0. with the Poisson correction method and 1000 bootstrap replicates and optimized with iTOL software.

The enhanced metabolization of insecticides by cytochrome P450 monooxygenases is a common insecticide resistance mechanism^16^. We identified 69 *cytochrome P450* genes that mapped to the *C. oryzae* chromosomes (Fig. 4). Phylogenetic analysis of P450s clearly represents four major clans, i.e., the CYP2, the CYP3, the CYP4, and the mitochondrial (Mito) clade (Fig. 4). The distribution of *P450* genes across the genome revealed 5 gene clusters with three or more *P450* genes (Supplementary Fig. 4).

### Thermal stress response

Thermal stress can alter the permeability of insects’ cell membranes, decrease their water content and affect their enzyme and protein activity. *Heat Shock Proteins* (*HSPs*) and antioxidant genes play important roles in protecting insects against these adverse consequences of thermal stress^17,18^. We manually annotated the *HSP* and antioxidant gene families in the *C. oryzae* genome, including 6 *HSP90*, 14 *HSP70*, 14 *HSP60*, 10 *HSP40*, 13 *small HSP* (*sHSP*), 2 *catalase* (*CAT*), 8 *peroxidase* (*POD*), 5 *superoxide dismutase* (*SOD*) and 22 *glutathione-S-transferase* (*GST*) (Supplementary Data 1; Supplementary Fig. 5–13). Supplementary Fig. 14 and 15 show the distribution of *HSP* genes and antioxidant genes, respectively, across the genome.

To investigate the role of stress response and antioxidant genes in ameliorating the adverse effects of heat stress in *C. oryzae*, we conducted a comparative transcriptomic analysis to identify genes that were differentially expressed in larvae that had been exposed to either normal, or high, temperatures. All 41,064 assembled unigenes were submitted to BLASTX for annotation in the NR, NT, SwissProt, KEGG, KOG, Pfam and GO databases. Pairwise comparisons among different temperature treatment groups showed that 1519 transcripts were upregulated and 1823 downregulated, between 24 °C and 33 °C (24 °C as control group), that 1487 transcripts were upregulated and 6996 downregulated, between 24 °C and 39 °C (24 °C as control group), and that 1641 transcripts were upregulated and 6232 downregulated, between 33 °C and 39 °C (33 °C as control group) (Supplementary Data 2). All differentially expressed annotated genes were classified into three categories, “Biological process”, “Cellular component” and “Molecular function”, by Gene ontology (GO) analysis. “Cellular process” and “Metabolic process” were the most common subcategories in the “Biological process” category, whereas “Binding” and “Catalytic activity” were the most common subcategories in the “Molecular function” category (Supplementary Fig. 16–19).

**Fig. 5:**
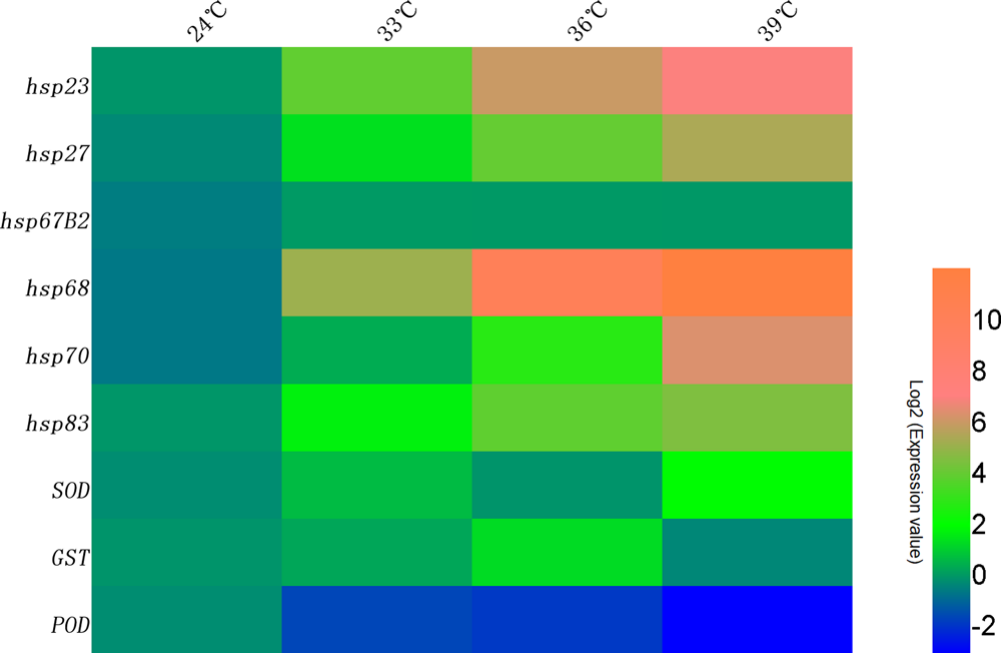
Effects of temperature stress on mRNA levels of stress and antioxidant genes in *Chlorops oryzae* larvae. mRNA levels were determined by qRT-PCR. Each gene had three biological replicates. The heat map was generated from log2-transformed expression ratios relative to the mRNA expression of stress and antioxidant genes in the 24 °C control group. Blue: low expression levels. Red: high expression levels.

We identified 62 candidate *HSPs*, 2 candidate *CAT*, 27 candidate *GST*, 10 candidate *POD* and 9 candidate *SOD*, genes in the different temperature treatment transcriptomes (Supplementary Fig. 20–24). We used qRT-PCR to validate RNA sequencing (RNA-seq) data by measuring the expression of stress response, and antioxidant, genes. The expression of antioxidant genes such as *SOD*, *GST* and *POD* was significantly affected by temperature (Fig. 5 and Supplementary Fig. 25), suggesting that these genes are involved in the response of *C. oryzae* to high temperature stress. Consistent with the RNA-seq results, some *HSP* genes, such as *HSP83*, *HSP70*, *HSP68*, *HSP67B2*, *HSP27* and *HSP23*, were also upregulated (Fig. 5 and Supplementary Fig. 25).

### Reproductive development

**Fig. 6:**
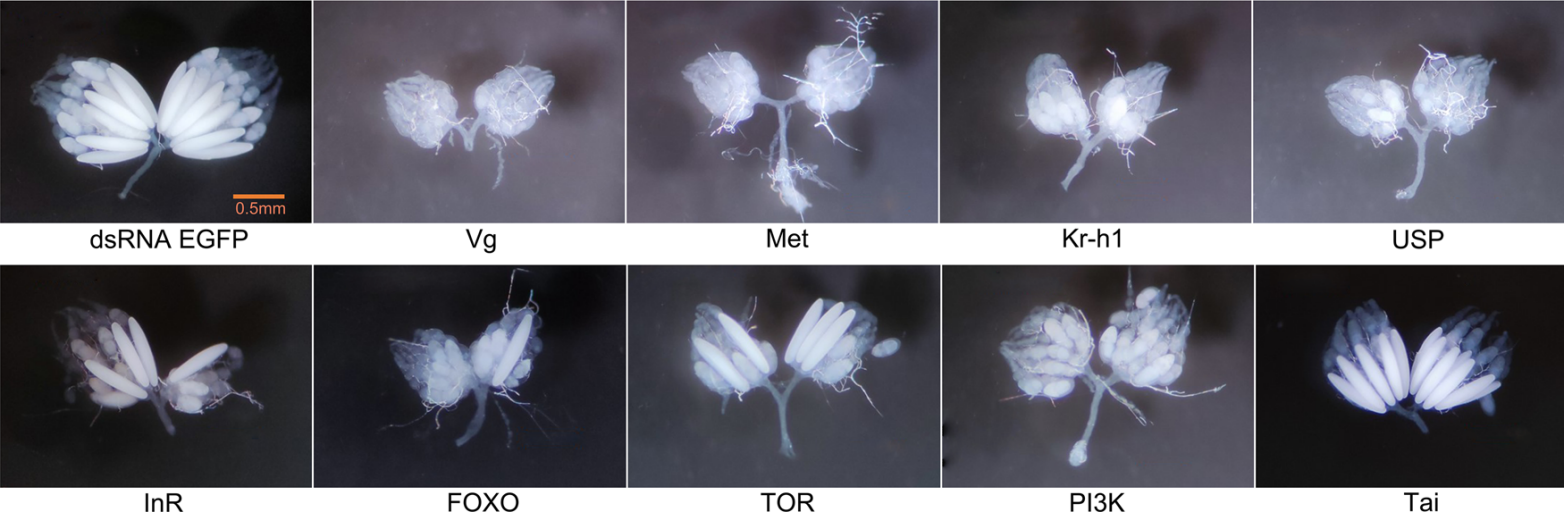
Effects of RNAi knockdown of the *Vitellogenin* (*Vg*) and eight other genes involved in the JH, 20E and insulin-like peptide, signaling pathways, on ovarian development. The control group was treated with an equivalent dose of dsEGFP. Scale bar, 0.5 mm. *Met*, *Kr-h1* and *Tai* are genes in the JH signaling pathway. *InR*, *FOXO*, *TOR* and *PI3K* are genes in the insulin signaling pathway. *USP* is gene in the 20E pathway.

It is well known that the reproductive capacity of insects is critical to insect population outbreaks. Ovarian maturity is fundamental for female insect reproduction, which can be regulated by juvenile hormone (JH), 20-ecdysterone (20E) or insulin through regulating vitellogenesis^19^. We performed RNAi experiments to disrupt JH, 20E and insulin-like, signaling in newly emerged adult females, focusing on how ovarian development is regulated by different upstream signals. RNAi knockdown of *vitellogenin* (*Vg*) completely prevented ovary maturation (Fig. 6 and Supplementary Fig. 26). Furthermore, RNAi knockdown of key genes in the JH pathway (*Methoprene-tolerant* (*Met*) and *Krüppel homolog 1* (*Kr-h1*)) reduced yolk deposition, thereby inhibiting ovarian development, but RNAi knockdown of *Taiman* (*Tai*, a binding partner of *Met*) had no effect on ovarian development (Fig. 6 and Supplementary Fig. 26). RNAi knockdown of key genes in the insulin pathway (*InR* (insulin receptor), *FOXO* (Forkhead box-containing protein)), *TOR* (Target of Rapamycin), and *PI3K* (phosphatidylinositol 3-kinase) and *USP* (ultraspiracle protein) (a key gene in the 20E pathway) also affected oocyte maturation and prevented ovarian development (Fig. 6 and Supplementary Fig. 26). These results indicate that JH, insulin, and 20E are crucial for normal ovary maturation in *C. oryzae*.

## Discussion

We generated a high-quality *C. oryzae* genome assembly by combing the PacBio Sequel system and HiSeq X Ten platform with Hi-C technology. Long-read sequencing and Hi-C assisted assembly strategies have previously produced high-quality genome assemblies of other animals^20,21^ and plants^22,23^. Our results, including contig N50, Scaffold N50, GC content, BUSCO evaluation and the full-length transcripts, indicate that the reference genome had high levels of completeness and accuracy and may provide a foundation for further genetic research on *C. oryzae*.

Cytochrome P450 is an ancient and large superfamily involved in the metabolism of exogenous and endogenous compounds^24^. This gene family has been well studied in insects due to its contribution to adaptation to exogenous compounds and pesticide resistance^21,25^. In some orders, the number of these detoxification family genes is related to the level of insecticide resistance. For example, compared to other blattodea species, the expanded *cytochrome P450 monooxygenase* gene family of American cockroaches *Periplaneta americana* is associated with higher insecticide resistance and survival under extreme conditions^26^. We predicted fewer *P450* genes in *C. oryzae* than in other Diptera. However, since other studies have found no obvious link between the size of detoxification gene families and resistance, *C. oryzae* may not have any less detoxification capacity than other insects and size could be corelated with the breadth of the host range^27^. Further studies are required to identify key genes in the *C. oryzae cytochrome P450* family.

Heat shock proteins are ubiquitous and evolutionarily conserved families of proteins in all living organisms that are critical for environmental adaptation^28^. HSPs usually act as molecular chaperones, facilitating the correct refolding of proteins and preventing the aggregation of denatured proteins, but they also participate in diverse cellular processes such as signal transduction, DNA replication, metabolic detoxification and immune defense reactions^29^. Furthermore, *HSPs* can be induced by extreme temperatures, oxidation, UV and heavy metals to help organisms withstand adverse environmental conditions^28^. Since their discovery in *D. melanogaster* larvae, HSPs have been found to be involved in the heat stress responses of many insects^30,31^. Our results show that the expression of several *HSPs* (*HSP83*, *HSP70*, *HSP68*, *HSP67B2*, *HSP27* and *HSP23*) was significantly upregulated in the high temperature treatment groups relative to the control, which suggests that these genes play an important role in counteracting heat stress in *C. oryzae*.

*HSP83* is a member of HSP90 family. HSP90 proteins often regulate the ability of animals to adapt to adverse environmental conditions and serve as the primary self-protection mechanism. Consistent with our results, *HSP83* was upregulated in *Sesamia nonagrioides* after exposure to an elevated temperature (40 °C)^32^. The others, *HSP70*, *HSP68* and *HSP67B2* belong to the most conserved *HSP* gene family-the *HSP70* family^31,33^. Several studies have found that *HSP70s* play important roles in resistance to heat stress. In *Drosophila*, thermotolerance was significantly improved by the inducible expression of *HSP70s*^33^. Furthermore, the survival rate of female *B. tabaci* subject to heat stress dramatically decreased after *HSP*70 knockdown^34^ and in *Diaphorina citri HSP70* was significantly upregulated in insects subject to heat stress^18^. Besides that, *HSP27* and *HSP23* are members of the *sHSP* family, which has the most diverse functions among stress-response proteins. Consistent with the results of a study on *Chironomus riparius*^35^, we found that expression of *HSP27* and *HSP23* markedly increased at high temperatures. Apart from responding to heat and oxidative stress, sHSPs may also be involved in diapause^36^, embryo formation, physiological regulation^37^ and metamorphosis^38^. Overall, our results indicate that *HSP83*, *HSP70*, *HSP68*, *HSP67B2*, *HSP27* and *HSP23* play important roles in the ability of *C. oryzae* to tolerate thermal stress. Further research is, however, required to determine the specific roles of these genes in *C. oryzae*.

Heat stress causes a variety of physiological stress responses in insects, including increased production of reactive oxygen species (ROS) that can cause oxidative damage. Oxidative damage in proteins ranges from specific amino acid modifications and peptide breakage to the loss of enzyme activity^39^. To prevent such damage, organisms have developed antioxidant defense mechanisms, such as specific antioxidant systems (e.g., vitamins, glutathione, antioxidant enzymes, etc.)^40^. Our results show that exposure to high temperatures significantly affected the expression of *SOD*, *GST* and *POD*, which suggests that these antioxidant enzymes are involved in antioxidant responses to thermal stress in *C. oryzae*. SOD is known to play an important role in reducing the level of superoxide radicals induced by low, or high, ambient temperatures^41^. In *Monochamus alternatus*, the *SOD* gene was upregulated in larvae exposed to 40 °C^17^. Similarly, we found that *SOD* expression was significantly higher in the 39 °C treatment group than in the 24 °C control group, suggesting that *SOD* was induced by exposure to high temperatures. *SOD* scavenges superoxide anions thereby protecting insects from thermal stress. GST is thought to participate in the inactivation of accumulated toxic, lipid, peroxidation products caused by oxidative damage and xenobiotics treatment^42^. We found that expression of *GST* was significantly higher in the 36 °C treatment group than in the control, and similar results have been reported in *Ostrinia furnacalis*^43^ and *Panonychus citri*^44^. Although *GST* was not significantly upregulated in the 39 °C treatment group compared to the control, this could be because lipid peroxidation was mitigated by other antioxidant mechanisms. In addition to SOD and GST, insects also have POD, which breaks down H_2_O_2_^45^. We found that *POD* expression was significantly lower in the temperature treatment groups relative to the control. Conversely, exposure to a high temperature (35 °C) for 1 h dramatically increased POD activity in *Aphidius gifuensis*^46^, whereas there was no significant difference in *POD* expression between high temperature treatment groups and the control (25 °C) in *P. citri*, even after the duration of exposure to high temperatures was increased^44^. Further research is required to understand the role of *POD* in the responses of insects to thermal stress.

Ovarian development, the most important part of the reproductive system of female insects^19^, is essential for maintaining insect populations. Ovarian maturity can be regulated by JH, 20E or insulin through regulating vitellogenesis^19^. Our RNAi experiments on newly emerged females demonstrate that JH, insulin and 20E are critical to the regulation of oocyte maturation and ovarian development in *C. oryzae*. Apparently, vitellogenesis and egg maturation are coordinated by three hormonal signals in this pest, which is not unexpected given the complex reproductive processes in dipterans^19^. And it also appears to be the case in *P. americana*^26^. These results highlight the importance of understanding the molecular mechanisms underlying hormonal signaling pathways during ovarian maturation. JH, which acts via *Met*, controls vitellogenesis and oocyte maturation. Knockdown of *Met* consequently inhibits JH-induced *Vg* expression, ovarian development and lipid accumulation^47^. As an early response gene in the JH signaling pathway, *Kr-h1* has been confirmed to play an important role in yolk formation and ovarian development in *Bactrocera dorsalis*, *Locusta migratoria* and *Helicoverpa armigera*^47–49^. Consistent with these findings, our results show that *Met* and *Kr-h1* RNAi depletion block ovarian maturation in *C. oryzae*. Similarly, knockdown of the endoplasmic reticulum glucose-regulated chaperone *Grp78* gene, which is also regulated by JH, significantly inhibited follicular cell development and reproduction in *L. migratoria*^50^. These findings show that the JH signaling pathway regulates insect reproduction via multiple factors.

We found that knockdown of key genes in the insulin pathway (*InR*, *FOXO*, *TOR*, and *PI3K*) decreased yolk deposition and blocked ovarian development. The insulin signaling pathway is dependent on adequate nutrition, only female mosquitoes that have obtained a blood-meal can complete normal ovarian development. After a blood meal, amino acids activate TOR signaling, phosphorylate transcription activator S6K and transcription inhibitor 4E-BP, and RNAi *Rheb*, S6K-mediated gene, blocks Vg expression and egg maturation^51^. TOR signaling often regulates insect reproduction in combination with insulin signaling. Insulin binds to the insulin receptor InR, inducing phosphorylation of *InR* and interacting with the substrate to activate the PI3K pathway after which the normal transcription of *Vg* is regulated by cascade phosphorylation (PDK), protease B (Akt/PKB) and FOXO^52^. RNAi-mediated silencing of *InR* has been found to have negative effects on insect reproduction in several species, confirming the role of the insulin pathway in controlling reproductive processes^53,54^. In addition to the direct effect of the insulin pathway on vitellogenesis, an interaction between this and other pathways may also regulate ovarian development in *C. oryzae*. In some insects, insulin/TOR signaling regulates ovarian maturation by affecting JH signaling or JH biosynthesis^55,56^. In adult *Drosophila*, *InR* mutations led to a decrease in JH titer^57^, whereas silencing *TOR* and starvation caused a significant decrease in the levels of JH synthase and JH synthesis mRNA in the corpora allata of adult female cockroaches^58^. The TOR nutritional signaling pathway was found to have a similar effect on JH biosynthesis in *A. aegypti* and *N. lugens*^59,60^. In *Blattella germanica*, knockdown of *InR* was found to inhibit JH biosynthesis and decrease *Vg* expression, thereby blocking ovarian development^53^. The regulatory networks controlling ovarian maturation in insects are clearly complicated, and additional research is therefore required to understand the molecular mechanisms controlling this process in *C. oryzae*.

*C. oryzae* outbreaks frequently in recent years. Adaptation to warmer temperatures may have increased the frequency of outbreaks. In addition, pesticide resistance also facilitates *C. oryzae* outbreaks by making the species more difficult to control. Reproductive development is also crucial for maintaining insect populations. In summary, this paper provides insights on possible reasons for *C. oryzae* frequent outbreaks in recent years. Our chromosome-level genome assembly should both facilitate future genetic research on the causes of *C. oryzae* outbreaks and support the development of sustainable control strategies for this pest.

## Methods

### Insects

*C. oryzae* larvae were collected in 2019 in Hanshou County, Hunan province, China, and reared on fresh rice stems in the laboratory. Larvae were kept at 24 ± 1 °C and >80% relative humidity, under a photoperiod of 16:8 (L:D) h. Larvae were used for Illumina sequencing for transcriptome analysis, and 100 female adults were collected for Illumina, PacBio, and Hi-C sequencing for genome analysis.

### Genome sequencing

The whole genome was sequenced on the PacBio Sequel System (https://www.pacb.com/products-and-services/pacbio-systems/sequel/) based on single-molecule real-time (SMRT) sequencing technology. The template library was constructed using a SMRTbell Template Prep Kit 1.0 and a SMRTbell Damage Repair Kit. Following the procedure described in the PacBio brochure “>20 kb Template Preparation Using BluePippin^™^ Size-Selection System (15–20 kb Cutoff) for Sequel^™^ Systems”, the quality DNA was fragmented with g-TUBE (covaries, 520079), concentrated with AMPure^®^ PB magnetic beads and the fragments eluted with Pacific Biosciences^®^ Elution Buffer. The fragments were damage-repaired with ExoVII, end-repaired with End Repair Mix and ligated with the blunt adapter. After removing failed ligation products with ExoIII and ExoVII, ligation products were purified twice with AMPure^®^ PB Beads, and selected for size with the BluePippin^™^ Size-Selection System. The fragments obtained were bead-purified, damage-repaired, and used as ~20 kb SMRTbell templates. These templates were annealed with primers and bound to DNA polymerase using a PacBio DNA/Polymerase Kit and magnetic beads, and loaded into the PacBio Sequel™ System for sequencing.

### Generation of short reads for genome correction

In order to collect Illumina paired-end reads, we used agarose electrophoresis (1% agarose gels) to check for possible degradation and contamination of genomic DNA, determined its purity with a NanoPhotometer® (IMPLEN, CA, USA) and measured its concentration with a Qubit^®^ 2.0 Fluorometer (Life Technologies, CA, USA). Only genomic DNA that passed these quality controls was included in the short fragment library constructed following the TruSeq DNA Sample Preparation Guide (Illumina, 15026486 Rev. C). This procedure mainly included the steps of DNA fragmentation, end-repairing, base “A” tailing, ligation of adapters, the recovery of DNA of the required size from gels and PCR amplification of the recovered DNA. Amplification products were used as libraries for sequencing once they passed quality checks. In brief, amplification products were quantified with Qubit2.0 and their size range determined with Agilent 2100. If fragments were within the expected size range, the library was accurately quantified with a Bio-RAD CFX 96 real time quantitative PCR thermocycler and a Bio-RAD KIT iQ SYBR GRN Q-PCR thermocycler. The quality library was sequenced on a HiSeq X Ten Platform set to the PE150 program and paired-end reads obtained.

### K-mer analysis

Before genome assembly, genome features can be estimated from the sequences obtained by sequencing. We used the analysis method based on K-mer to estimate the genome size. We iteratively selected the sequence with the length of K bases from a continuous sequence. If the length of each sequence is L, the length of K-mer is K, then L-K + 1 K-mer can be obtained. Here, we took *K* = 21 for analysis. The distribution of K-mers depends on the characteristics of the genome and follow a Poisson’s distribution.

### Genome assembly

The quality of the reads exported by Sequel™ Systems was evaluated with the in-built High Quality Region Finder (HQRF), which identifies the longest high quality region generated for each read by a singly-loaded DNA polymerase according to the signal to noise ratio. Upon generation by the system, all bases were marked with “!” in order to perfect the format. High-quality reads (or regions) were marked with “0.8” and low-quality ones with “0”.

High-quality reads were assembled into contigs using Canu (v1.5 https://github.com/marbl/canu) by setting the parameters as follows: canu -pacbio-raw sample.subreads.fasta -p sample -d sample-canu genomeSize = 40 m gridEngineMemoryOption = “-l vf = MEMORY”. Canu used all-versus-all overlap information to correct individual reads. It selected these overlaps in a two-step filtration process comprised of global and local filtration. Global filtration identified targets where a read may provide correction support, whereas local filtration allowed a read to accept, or reject, the correction evidence provided by other reads. At the trimming stage, Canu identified the region of each read without correction support, trimming or splicing reads into their longest regions with correction support. These regions were subject to a final check for sequencing errors, and then used to construct the best overlap graph based on the output contigs and to compile summary statistics.

Errors in the primary assembly were identified and corrected with BLASR (v5.1, https://github.com/ Pacific Biosciences/blasr) and Arrow (v2.2.1), a tool built in Smrt Link (https://downloads.pacbcloud.com/public/software/installers/smrtlink_5.0.1.9585.zip). The PacBio reads were first mapped to the raw contigs using BLASR with the parameters:—bam—bestn 5—minMatch 18—nproc 4—minSubreadLength 1000—minAlnLength 500—minPctSimila rity 70—minPctAccuracy70—hitPolicy randombest—randomSeed 1, after which consensus sequences and variant calls were obtained *via* Arrow (v2.2.1) with the default parameters.

The consensus genome was subject to a final round of base-error correction (polishing) by referring to the Illumina reads with BWA (v0.7.9a) and Pilon (v1.22, https://github.com/broadinstitute/pilon). The Illumina paired-end reads were mapped to the contigs by BWA (parameter, -k 30), after which Pilon (v1.22) (default parameters) used this alignment to correct the assembly. The quality of the genome sequence obtained was further evaluated with BUSCO (v3.0.1, http://busco.ezlab.org/) (default parameters) based on a set of single-copy orthologous eukaryotes genes.

### Hi-C

We performed Hi-C sequencing to facilitate assembly of the *C. oryzae* genome. After crosslinking, samples were used for quality control. The Hi-C library was then prepared and sequenced on the Illumina Novaseq platform with 2 × 150 bp reads at Annoroad Gene Technology Co. Ltd. (Supplementary Table 10; Supplementary Fig. 27). We first used the bowtie 2 end-to-end algorithm to align cleaned reads with the reference genome^61^. Unmapped reads mainly consisted of chimeric fragments that spanned the ligation junction. According to the Hi-C introduction and fill-in strategy, HiC-Pro (v2.7.8) was used to detect the ligation site with an exact matching program and to align the 5' segment read on the genome^62^. The results of each mapping step were then merged into a single alignment file. Lachesis, the assembly package, was used to cluster, order and orient reads. Finally, we cut the chromosomes predicted by Lachesis into equal-length bins, such as 1 Mb or 500Kb, and constructed a heat map according to the interaction signals revealed by the effective mapping between bins (Supplementary Fig. 27).

### Genome annotation

Two methods, homologous sequence prediction and ab initio prediction, were used to predict repetitive sequences. Homologous sequence prediction is based on RepBase (https://www.girinst.org/server/RepBase/index.php), a repeat sequence database. RepeatMasker and RepeatProteinMask were used to predict sequences similar to known repeat sequences^63^. RepeatModeler (http://www.repeatmasker.org/RepeatModeler/) was used in ab initio prediction. First, a *de novo* repeat sequence library was established by RepeatModeler, and then repeat sequences were predicted by RepeatMasker. In addition, the ab initio prediction method was also used to find tandem repeat sequences in the genome with TRF software^64^.

Gene structure prediction was performed using three strategies: evidential support of transcriptional data, homologous prediction, and ab initio prediction. For the evidential support of transcriptional data, we used EST/CDA sequence and genome alignment to predict gene structure, with the commonly used software PASA (http://pasa.sourceforge.net/)^65^. In homologous prediction, the coding protein sequences of known homologous species (*Drosophila melanogaster*, *Ceratitis capitata*, and *Lucilia cuprina*) were compared with genome sequences of *C. oryzae* and the gene structure was predicted by BLAST (http://blast.ncbi.nlm.nih.gov/Blast.cgi)^66^, Genewise (http://www.ebi.ac.uk/~birney/wise2/)^67^. Software based on the statistical characteristics of genomic sequence data (such as codon frequency, exon-intron distribution) was used to predict gene structure in ab initio prediction. The most commonly used software packages are Augustus (http://augustus.gobics.de/), SNAP (https://github.com/KorfLab/SNAP) and GeneMark (http://exon.gatech.edu/GeneMark/). Finally, to synthesize the above forecast results, the gene sets predicted by each strategy were integrated into a non-redundant and more complete gene set with EVidenceModeler (EVM) (http://evidencemodeler.github.io/)^68^.

Functional annotation of genes was performed based on the best match to the Swissprot (https://web.expasy.org/docs/swiss-prot_guideline.html), NT (https://www.ncbi.nlm.nih.gov/nucleotide/), NR (ftp://ftp.ncbi.nlm.nih.gov/blast/db/FASTA/nr.gz), PFAM (http://xfam.org/), eggNOG (http://eggnogdb.embl.de/), GO (http://geneontology.org/page/go-database) and KEGG (http://www.genome.jp/kegg/), database. For the final integration, information from different functional annotation sources were combined for each gene.

Non-coding RNA, including ribosome RNA (rRNA), small nuclear RNA (snRNA), microRNA (miRNA) and transfer RNA (tRNA) were identified. rRNA, snRNA, and miRNA were predicted by comparison with known noncoding RNA libraries, Rfam (http://rfam.xfam.org/), and tRNA was annotated using the tRNAscan-SE (http://lowelab.ucsc.edu/tRNAscan-SE/) software package^69^.

### Orthology and phylogeny

In addition to *C. oryzae*, 14 representative insect species: *Zootermopsis nevadensis* (accession number: GCA_000696155.1) (Isoptera, outgroup), *Nilaparvata lugens* (downloaded from OMIGA annotation project) and *B. tabaci* (accession number: GCF_001854935.1) (Hemiptera), *Apis mellifera* (accession number: GCF_003254395.2) and *Nasonia vitripennis* (accession number: GCF_000002325.3) (Hymenoptera), *Tribolium castaneum* (accession number: GCF_000002335.3) and *Anoplophora glabripennis* (accession number: GCA_000390285.1) (Coleoptera), *Bombyx mori* (accession number: GCF_000151625.1) and *Chilo suppressalis* (downloaded from InsectBase v2.0) (Lepidoptera), *D. melanogaster* (accession number: GCF_000001215.4), *C. capitata* (accession number: GCF_000347755.1), *L. cuprina* (accession number: GCF_000699065.1), *Anopheles gambiae* (downloaded from VectorBase) and *Aedes aegypti* (accession number: GCF_002204515.2) (Diptera), were selected for orthology analysis. Protein sequences translated from the longest transcripts of each gene were aligned to identify conserved orthologs with BLASTP (*E*-value = 1e-5). Finally, OrthoMCL was used to cluster gene families based on the BLASTP results^70^.

We used protein sequences of identified single-copy genes to reconstruct the phylogeny and MUSCLE (v3.8.31) (http://www.drive5.com/muscle/) to perform multiple alignment of the protein sequence of each orthologous group. Phylogenetic analysis in PhyML (v3.0) was performed using the maximum likelihood methods with 100 bootstrap replicates^71^. The mcmctree (http://abacus.gene.ucl.ac.uk/software/paml.html) (burn-in = 20,000, sample-frequency = 2) in PAML (v4.9) packagewas used to estimate divergence time with the BRMC method^72^. Calibration time from the TimeTree (http://www.timetree.org/) was used to calibrate divergence time.

### Gene family expansion and contraction analysis

According to the cluster analysis results of gene families, filtering them to remove gene families whose number of genes is >200 in one species and <2 in other species, and gene families whose total number of genes in the gene family is less than the number of species family. Then, using the CAFÉ software (http://sourceforge.net/projects/cafehahnlab) with PGM (probabilistic graphical models) model to simulate the acquisition and loss of genes under the specified evolutionary tree, and analyzing the expansion and contraction of gene family through hypothesis test.

### Gene family identification and analysis

We first downloaded a set of reference protein sequences from NCBI GenBank and used Hidden Markov models (HMMs) to obtain references for gene identification. SPDE (v1.2)^73^ (*E*-value ≤ 1e-5) was used to search for candidate genes in the *C. oryzae* genome. Then, genes were manually annotated by BLASTP and GENEWISE, and the number of genes is consistent with that the number we identified. 62 genes are complete and 7 genes needed to be fixed. After which a neighbor-joining tree was constructed using MEGA7^74^ with the Poisson correction method and 1000 bootstrap replicate searches. The final phylogenetic tree was prepared in iTOL (v5) (http://itol.embl.de) and Adobe Illustrator (Adobe Systems, San Jose, CA, USA). The phylogenetic tree of the target gene family was constructed using genes from *C. oryzae*, *D. melanogaster*, *L. cuprina*, and *C. capilata* (Supplementary Data 3).

### Location of P450 genes, HSP genes and antioxidant genes on the chromosome

To locate all identified genes on the chromosome, we first used SPDE (v1.2)^73^ (*E*-value ≤ 1e-5) to search for candidate genes in the *C. oryzae* genome and mapped those found onto the chromosome using a GFF3 file and TBtools (v1.071)^75^.

### Transcriptome sequencing and analysis during larval temperature stress experiments

*C. oryzae* causes economically damage to rice crops and can complete second and third generations under high temperatures, which can result in outbreaks. To understand the responses of *C. oryzae* larvae to temperature stress, larvae were randomly assigned to one of three temperature treatment groups: 33 °C, 36 °C and 39 °C. Each group was comprised of 20 larvae and had three biological replicates. Larvae in each group were subjected to one of the above temperature treatments for 2 h whereas the control group was kept at 24 °C. At the end of the experiment larvae were frozen in liquid nitrogen for 5 min, then stored at −80 °C until required. 1.5 μg of RNA from each sample was used to construct a cDNA (Complementary DNA) library. An RNA-seq library was sequenced on an Illumina Hiseq platform. SOAPnuke, a self-developed filtering software, was used to compile statistics, and raw reads were cleaned by filtering them against reads containing adapters, poly-N and low-quality reads with trimmomatic. *De novo* assembly of clean reads (the removal of PCR duplicates to improve assembly efficiency) was conducted using Trinity, after which the assembled transcripts were clustered and de-duplicated using Tgicl to obtain Unigenes. Clean reads were aligned to the genomic sequence with Bowtie 2^61^, after which the gene expression level of each sample was calculated using RSEM^76^. The DEGseq method is based on the Poisson distribution. DEGs were detected using the method described in Wang, Feng, Wang, Wang, and Zhang (2010)^77^. *P*-values were adjusted using Benjamini and Storey’s approach to control false positives. Genes with a ≥ 2-fold difference in expression, and an adjusted *P*-value ≤ 0.001, were considered significantly differentially expressed.

### RNA interference

RNAi was used to determine the function of target genes in oocyte maturation using the *EGFP* (*enhanced green fluorescent protein*, GenBank Accession No. U55762) gene as a parallel control. For double-strand RNA (dsRNA) preparation specific primers (Supplementary Data 4) conjugated with the T7 promoter sequence were first used for PCR amplification, and the resultant PCR products were used as templates for dsRNA synthesis. dsRNA was synthesized using the T7 RiboMAX Express RNAi System (Promega, Madison, WI, United States) according to the manufacturer’s instructions. 500 ng dsRNA was injected into each newly emerged adult female in the treatment group and the same dosage of dsEGFP injected into those in the control group. Three biological replicates were performed. The ovarian morphology of females in each group was observed under a stereomicroscope (Motic SMZ-161, Motic Group Co., Xiamen, China) 72 h after dsRNA injection.

### Total RNA extraction and quantitative real-time PCR

Total RNA was extracted using TRIzol reagent (Invitrogen, Carlsbad, CA, USA) according to the manufacturer’s instructions. RNA purity was verified with gel electrophoresis and its concentration measured with a Qubit^®^ RNA Assay Kit in Qubit® 2.0 Flurometer (Life Technologies, CA, USA). qPCR primers were designed using the NCBI profile server (http://www.ncbi.nlm.nih.gov/tools/primer-blast)(see Supplementary Data 4 for a list of the primers used). cDNA was synthesized using a PrimeScript RT Reagent Kit with gDNA Eraser (Perfect Real Time) (Takara, Dalian, China) according to the manufacturer’s instructions. cDNA templates were diluted 5 times with deionized water. qRT-PCR was performed on a CFX96 Touch^TM^ Real-Time PCR Detection System (Bio-Rad Laboratories, Hercules, CA, USA) in a reaction volume of 20 μl using TB Green^TM^ Premix Ex Taq^TM^ II (Takara), according to the manufacturer’s instructions. *RPS15* (*ribosomal protein S15*) and *RP49* (*ribosomal protein 49*) were the internal references genes^78^. A two-step program was performed as follows: 95 °C for 30 s, 40 cycles at 95 °C for 10 s and 59 °C for 30 s. A melting curve analysis was performed from 55 °C to 95 °C to determine the specificity of the qPCR primers and their efficiency was verified by calculating a standard curve (cDNA concentration vs. Ct) based on the dilution gradient of the templates. The 2^-ΔΔCt^ method was used to calculate the relative expression levels of target genes^79^.

### Statistics and reproducibility

Statistical analysis of the qRT-PCR results was conducted in GraphPad Prism 8 software (GraphPad Software Inc., San Diego, CA, United States). Data are presented as mean ± standard error (SE). The statistical significance of differences in the expression of genes among temperature treatment groups was analyzed with one-way analysis of variance (ANOVA) followed by Tukey’s honestly significant difference test for multiple sample comparisons (*P* < 0.05). The statistical significance of differences in gene expression between the RNAi treatment and control group was evaluated with Student’s *t*-test (^∗^*P* < 0.05, ^∗∗^*P* < 0.01, ^∗∗∗^*P* < 0.001). Go term analysis of upregulated pathways was performed using the OmicShare tools, a free online platform for data analysis (https://www.omicshare.com/tools)^80^.

### Reporting summary

Further information on research design is available in the Nature Research Reporting Summary linked to this article.

## Supplementary information

Supplementary Information

Description of Additional Supplementary Files

Supplementary Data 1

Supplementary Data 2

Supplementary Data 3

Supplementary Data 4

Reporting summary

## Data Availability

All raw data from the *Chlorops oryzae* genome have been deposited in the SRA under SRR14340331 (BioProject PRJNA728371). This Whole Genome Shotgun project has been deposited at DDBJ/ENA/GenBank under the accession JAIPUU000000000. The *C. oryzae* transcriptome data for genome annotation are also stored in the SRA (SRR7528441, SRR7528446, SRR7528467, SRR7529086, SRR7529100, SRR7533623, SRR7534236, SRR7534658 and SRR7534603) (BioProject PRJNA481388, PRJNA 481391, PRJNA 481407, PRJNA 481440, PRJNA 481449, PRJNA 481587, PRJNA 481589, PRJNA 481604, PRJNA 481612). The Hi-C data have been deposited in the NCBI GEO under the accession number GSE210874. All other relevant data are available upon request.

## References

[1] Takeda M (1998). Genetic basis of photoperiodic control of summer and winter diapause in geographic ecotypes of the rice stem maggot, *Chlorops oryzae*. Entomol. Exp. appl..

[2] Hirao J (1970). Comparative studies on the development of geographical populations from the 2- and 3-generation areas in the rice stem maggot, *Chlorops oryzae* Matsumura. Bull. Tohoku. Nat. Agric..

[3] Li F (2019). Insect genomes: progress and challenges. Insect Mol. Biol..

[4] Jiang F, Liang L, Wang J, Zhu S (2022). Chromosome-level genome assembly of *Bactrocera dorsalis* reveals its adaptation and invasion mechanisms. Commun. Biol..

[5] Adams MD (2000). The genome sequence of *Drosophila melanogaster*. Science.

[6] Holt RA (2002). The genome sequence of the Malaria Mosquito *Anopheles gambiae*. Science.

[7] Scott JG (2014). Genome of the house fly, *Musca domestica* L., a global vector of diseases with adaptations to a septic environment. Genome Biol..

[8] Papanicolaou A (2016). The whole genome sequence of the Mediterranean fruit fly, *Ceratitis capitata* (Wiedemann), reveals insights into the biology and adaptive evolution of a highly invasive pest species. Genome Biol..

[9] Pollard, E. & Yates, T. J. *Monitoring Butterflies for Ecology and Conservation* (Chapman & Hall, London,1993).

[10] Bale JS (2002). Herbivory in global climate change research: direct effects of rising temperature on insect herbivores. Glob. Chang. Biol..

[11] IPCC. *Climate Change 2013 -Quotations* (IPCC, 2014).

[12] Wang YJ, Zhou BT, Ren YY, Sun CH (2016). Impacts of global climate change on China climate security. J. Appl. Meteorol. Sci..

[13] Su H (2019). Comparative transcriptome profiling reveals candidate genes related to insecticide resistance of *Glyphodes pyloalis*. Bull. Entomol. Res..

[14] Liu N (2015). Insecticide resistance in mosquitoes: impact, mechanisms, and research directions. Annu. Rev. Entomol..

[15] Feyereisen, R. In *Comprehensive Molecular Insect Science* (Gilbert, L. I. et al.) 1–77 (Elsevier BV, Amsterdam, 2005).

[16] Ranson H (2002). Evolution of supergene families associated with insecticide resistance. Science.

[17] Li H (2020). Comparative transcriptome analysis of the heat stress response in *Monochamus alternatus* Hope (Coleoptera: Cerambycidae). Front. Physiol..

[18] Xiong Y (2019). Comparative transcriptome analysis reveals differentially expressed genes in the Asian citrus psyllid (*Diaphorina citri*) upon heat shock. Comp. Biochem. Physiol., Part D: Genomics Proteom..

[19] Roy S, Saha TT, Zou Z, Raikhel AS (2018). Regulatory pathways controlling female insect reproduction. Annu. Rev. Entomol..

[20] Li, Y. et al. Chromosome-level assembly of the mustache toad genome using third-generation DNA sequencing and Hi-C analysis. *GigaScience*10.1093/gigascience/giz114 (2019). 10.1093/gigascience/giz114 PMC6755253 31544214

[21] Wan FH (2019). A chromosome-level genome assembly of *Cydia pomonella* provides insights into chemical ecology and insecticide resistance. Nat. Commun..

[22] Schmidt MHW (2017). *De Novo* assembly of a new *Solanum pennellii* accession using Nanopore sequencing. Plant Cell.

[23] Wu H (2019). A high-quality *Actinidia chinensis* (kiwifruit) genome. Hortic. Res..

[24] Scott JG, Wen Z (2001). Cytochromes P450 of insects: the tip of the iceberg. Pest Manag. Sci..

[25] Wang H (2018). CYP6AE gene cluster knockout in *Helicoverpa armigera* reveals role in detoxification of phytochemicals and insecticides. Nat. Commun..

[26] Li S (2018). The genomic and functional landscapes of developmental plasticity in the American cockroach. Nat. Commun..

[27] Rane RV (2016). Are feeding preferences and insecticide resistance associated with the size of detoxifying enzyme families in insect herbivores?. Curr. Opin. Insect Sci..

[28] King AM, MacRae TH (2015). Insect heat shock proteins during stress and diapause. Annu. Rev. Entomol..

[29] Richter K, Haslbeck M, Buchner J (2010). The heat shock response: life on the verge of death. Mol. Cell.

[30] Guo X, Feng J (2018). Comparisons of expression levels of heat shock proteins (*hsp70* and *hsp90*) from *Anaphothrips obscurus* (Thysanoptera: Thripidae) in polymorphic adults exposed to different heat shock treatments. J. Insect Sci..

[31] Wang XR (2019). Genome-wide identification and characterization of HSP gene superfamily in whitefly (*Bemisia tabaci*) and expression profiling analysis under temperature stress. Insect Sci..

[32] Gkouvitsas T, Kontogiannatos D, Kourti A (2009). Expression of the *Hsp83* gene in response to diapause and thermal stress in the moth *Sesamia nonagrioides*. Insect Mol. Biol..

[33] Bettencourt BR, Hogan CC, Nimali M, Drohan BW (2008). Inducible and constitutive heat shock gene expression responds to modification of *Hsp70* copy number in *Drosophila melanogaster* but does not compensate for loss of thermotolerance in *Hsp70* null flies. BMC Biol..

[34] Lu ZC, Wan FH (2011). Using double-stranded RNA to explore the role of heat shock protein genes in heat tolerance in *Bemisia tabaci* (Gennadius). J. Exp. Biol..

[35] Raquel MF, Mercedes de la F, Gloria M, José-Luis MG (2015). Characterization of six small *HSP* genes from *Chironomus riparius* (Diptera, Chironomidae): Differential expression under conditions of normal growth and heat-induced stress. Comp. Biochem. Physiol., Part A: Mol. Integr. Physiol..

[36] Ponnuvel KM, Murthy GN, Awasthi AK, Rao G, Vijayaprakash NB (2010). Differential gene expression during early embryonic development in diapause and non-diapause eggs of multivoltine silkworm *Bombyx mori*. Indian J. Exp. Biol..

[37] Nguyen TM, Bressac C, Chevrier C (2013). Heat stress affects male reproduction in a parasitoid wasp. J. Insect Physiol..

[38] Gu J, Huang LX, Shen Y, Huang LH, Feng QL (2012). *Hsp70* and small Hsps are the major heat shock protein members involved in midgut metamorphosis in the common cutworm, *Spodoptera litura*. Insect Mol. Biol..

[39] Stadtman ER, Levine RL (2003). Free radical-mediated oxidation of free amino acids and amino acid residues in proteins. Amino Acids.

[40] Cossu C (1997). Glutathione reductase, selenium-dependent glutathione peroxidase, glutathione levels and lipid peroxidation in freshwater bivalves, *Unio tumidus*, as biomarkers of aquatic contamination in field studies. Ecotoxicol. Environ. Saf..

[41] Park MS, Jo PG, Choi YK, An KW, Choi CY (2009). Characterization and mRNA expression of Mn-SOD and physiological responses to stresses in the Pacific oyster *Crassostrea gigas*. Mar. Biol. Res..

[42] Qin G (2013). Characterization and functional analysis of four glutathione S transferases from the migratory locust, *Locusta migratoria*. PLoS ONE.

[43] Chen KK (2019). Transcription analysis of the stress and immune response genes to temperature stress in *Ostrinia furnacalis*. Front. Physiol..

[44] Yang LH, Huang H, Wang JJ (2010). Antioxidant responses of citrus red mite, *Panonychus citri* (McGregor) (Acari: Tetranychidae), exposed to thermal stress. J. Insect Physiol..

[45] Lee K (2005). Characterization of a silkworm thioredoxin peroxidase that is induced by external temperature stimulus and viral infection. Insect Biochem. Mol. Biol..

[46] Kang ZW (2017). The potential coordination of the heat-shock proteins and antioxidant enzyme genes of *Aphidius gifuensis* in response to thermal stress. Front. Physiol..

[47] Yue Y (2018). Involvement of Met and Kr-h1 in JH-mediated reproduction of female *Bactrocera dorsalis* (Hendel). Front. Physiol..

[48] Song J, Wu Z, Wang Z, Deng S, Zhou S (2014). Kruppel-homolog 1 mediates juvenile hormone action to promote vitellogenesis and oocyte maturation in the migratory locust. Insect Biochem. Mol. Biol..

[49] Zhang WN (2018). Dissecting the role of Kruppel homolog 1 in the metamorphosis and female reproduction of the cotton bollworm, *Helicoverpa armigera*. Insect Mol. Biol..

[50] Luo M (2017). Juvenile hormone differentially regulates two *Grp78* genes encoding protein chaperones required for insect fat body cell homeostasis and vitellogenesis. J. Biol. Chem..

[51] Roy SG, Raikhel AS (2011). The small GTPase Rheb is a key component linking amino acid signaling and TOR in the nutritional pathway that controls mosquito egg development. Insect Biochem. Mol. Biol..

[52] Sheng Z, Xu J, Bai H, Zhu F, Palli SR (2011). Juvenile hormone regulates vitellogenin gene expression through insulin-like peptide signaling pathway in the red flour beetle, *Tribolium castaneum*. J. Biol. Chem..

[53] Abrisqueta M, Suren-Castillo S, Maestro JL (2014). Insulin receptor mediated nutritional signalling regulates juvenile hormone biosynthesis and vitellogenin production in the German cockroach. Insect Biochem. Mol. Biol..

[54] Brown MR (2008). An insulin-like peptide regulates egg maturation and metabolism in the mosquito. Aedes aegypti. Proc. Natl Acad. Sci. USA.

[55] Tatar M (2001). A mutant *Drosophila* insulin receptor homolog that extends life-span and impairs neuroendocrine function. Science.

[56] Xu J, Sheng Z, Palli SR (2013). Juvenile hormone and insulin regulate trehalose homeostasis in the red flour beetle, *Tribolium castaneum*. PLoS Genet..

[57] Tu MP, Yin CM, Tatar M (2005). Mutations in insulin signaling alter juvenile hormone synthesis in *Drosophila melanogaster*. Gen. Comp. Endocrinol..

[58] Maestro JL, Cobo J, Bellés X (2009). Target of rapamycin (TOR) mediates the transduction of nutritional signals into juvenile hormone production. J. Biol. Chem..

[59] Lu K, Chen X, Liu WT, Zhou Q (2016). TOR pathway-mediated juvenile hormone synthesis regulates nutrient-dependent female reproduction in *Nilaparvata lugens* (Sta˚l). Int. J. Mol. Sci..

[60] Pérez-Hedo M, Rivera-Perez C, Noriega FG (2013). The insulin/TOR signal transduction pathway is involved in the nutritional regulation of juvenile hormone synthesis in *Aedes aegypti*. Insect Biochem. Mol. Biol..

[61] Langmead B, Salzberg SL (2012). Fast gapped-read alignment with Bowtie 2. Nat. Methods.

[62] Servant N (2015). HiC-Pro: an optimized and flexible pipeline for Hi-C data processing. Genome Biol..

[63] Tarailo-Graovac M, Chen N (2009). Using RepeatMasker to identify repetitive elements in genomic sequences. Curr. Protoc. Bioinf..

[64] Gary B (1999). Tandem repeats finder: a program to analyze DNA sequences. Nucleic Acids Res..

[65] Roberts A, Pimentel H, Trapnell C, Pachter L (2011). Identification of novel transcripts in annotated genomes using RNA-Seq. Bioinformatics.

[66] McGinnis S, Madden TL (2004). BLAST: at the core of a powerful and diverse set of sequence analysis tools. Nucleic Acids Res..

[67] Birney E, Clamp M, Durbin R (2004). GeneWise and genomewise. Genome Res..

[68] Haas BJ (2008). Automated eukaryotic gene structure annotation using EVidenceModeler and the program to assemble spliced alignments. Genome Biol..

[69] Lowe TM, Eddy SR (1997). tRNAscan-SE: a program for improved detection of transfer RNA genes in genomic sequence. Nucleic Acids Res..

[70] Li L, Stoeckert CJ, Roos DS (2003). OrthoMCL: identification of ortholog groups for eukaryotic genomes. Genome Res..

[71] Guindon S (2010). New algorithms and methods to estimate maximum-likelihood phylogenies: Assessing the performance of PhyML 3.0. Syst. Biol..

[72] Yang Z (2007). PAML 4: phylogenetic analysis by maximum likelihood. Mol. Biol. Evol..

[73] Xu D (2020). SPDE: A multi-functional software for sequence processing and data extraction. Bioinformatics.

[74] Kumar S, Stecher G, Tamura K (2016). MEGA7: molecular evolutionary genetics analysis version 7.0 for bigger datasets. Mol. Biol. Evol..

[75] Chen C (2020). TBtools: an integrative toolkit developed for interactive analyses of big biological data. Mol. Plant.

[76] Li B, Dewey CN (2011). RSEM: accurate transcript quantification from RNA-Seq data with or without a reference genome. BMC Bioinf..

[77] Wang L, Feng Z, Wang X, Wang X, Zhang X (2010). DEGseq: an R package for identifying differentially expressed genes from RNA-seq data. Bioinformatics.

[78] Tian P (2019). Evaluation of appropriate reference genes for investigating gene expression in *Chlorops oryzae* (Diptera: Chloropidae). J. Econ. Entomol..

[79] Livak KJ, Schmittgen TD (2001). Analysis of relative gene expression data using real-time quantitative PCR and the 2^-ΔΔCT^ method. Methods.

[80] Qie C (2020). Single-cell RNA-Seq reveals the transcriptional landscape and heterogeneity of skin macrophages in Vsir^-/-^ murine psoriasis. Theranostics.

