# Supplementary material for: A chromosome-level genome assembly provides insights into the environmental adaptability and outbreaks of Chlorops oryzae
Source: Commun Biol. 2022 Aug 26;5:881. doi: 10.1038/s42003-022-03850-7 (PMC9418232; doi:10.1038/s42003-022-03850-7)
Supplement: Supplementary file 1 — Supplementary Information [file 42003_2022_3850_MOESM1_ESM.pdf]

Supplementary Information

A chromosome-level genome assembly provides insights into the environmental adaptability and outbreaks of *Chlorops oryzae*

Ailin Zhou[1, 2], Cong Huang[3], Yi Li[4], Xinwen Li[4], Zhengbing Zhang[4], Hualiang He[1], Wenbing Ding[1, 2], Jin Xue[1], Youzhi Li[1, 2*] and Lin Qiu[1*]

[1]Hunan Provincial Key Laboratory for Biology and Control of Plant Diseases and Insect Pests, College of Plant Protection, Hunan Agricultural University, Changsha 410128, China

[2]Hunan Provincial Engineering & Technology Research Center for Biopesticide and Formulation Processing, Changsha 410128, China

[3]Shenzhen Branch, Guangdong Laboratory for Lingnan Modern Agriculture, Genome Analysis Laboratory of the Ministry of Agriculture, Agricultural Genomics Institute at Shenzhen, Chinese Academy of Agricultural Sciences, Shenzhen 518120, China

[4]Plant Protection and Inspection Station, Agriculture and Rural Development of Hunan Province, Changsha 410005, China

[*]Corresponding author e-mail: Youzhi Li: liyouzhi@hunau.edu.cn; Lin Qiu: qiulin@hunau.edu.cn

Supplementary Tables

Supplementary Table 1. Genome characteristics statistic of *Chlorops oryzae*.

Sample	*Chlorops oryzae*
K-mer	21
K-mer number	46,112,520,590
K-mer depth	97
Genome size (Mbp)	475.3900
Revised genome size (Mbp)	462.7600
Heterozygous ratio (%)	0.6800
Repeat (%)	43.9000

Supplementary Table 2. Summary of *Chlorops oryzae* genome assembly.

Category	Contigs (bp)	Scaffolds (bp)
Total length	447,596,911	447,780,111
Max length	14,372,090	173,276,640
N50	1,171,122	117,565,011
N60	826,533	117,565,011
N70	522,786	97,042,877
N80	264,365	97,042,877
N90	50,862	29,535,905
Total sequence numbers	3,407	1,575

Supplementary Table 3. BUSCO integrity assessment of *Chlorops oryzae* genome.

Items	Number	Percent (%)
Complete BUSCOs (C)	1,024	96.1
Complete and single-copy BUSCOs (S)	1,002	94.0
Complete and duplicated BUSCOs (D)	22	2.1
Fragmented BUSCOs (F)	7	0.7
Missing BUSCOs (M)	35	3.2
Total BUSCO groups searched	1,066	100

Supplementary Table 4. Statistics on full-length transcripts mapped to the *Chlorops oryzae* reference genome.

Feature	Number
Clean Reads	372,815,028
Clean Bases	55,922,254,200
Mapped Reads	362,838,484
Mapped Reads Rate (%)	97.32
Mapped Bases	52,802,201,413
Mapped Bases Rate (%)	94.42
Mean Depth	117.56
Coverage Rate (%)	96.76

Supplementary Table 5. Classification of repeat sequences identified in the *Chlorops oryzae* genome.

Repeat types	Length occupied (bp)	Percentages of sequence (%)
DNA	18,570,151	7.25
LINE	11,389,348	5.03
SINE	33,009	0.01
LTR	18,988,274	9.05
Unknown	2,763,580	33.16
Other	7,763,736	5.50
Total	56,303,880	56.30

Supplementary Table 6. The evidence of gene prediction in *Chlorops oryzae* genome.

Gene set	Number of genes	Average gene length (bp)	Average cds length (bp)	Average exons per gene	Average exon length (bp)	Average intron length (bp)
Augustus	15295	14105.36	1532.70	4.06	377.07	4103.29
GeneMark	20041	6808.55	1218.88	4.63	263.31	1541.24
SNAP	47556	6795.44	615.71	3.86	159.38	2159.29
Ceratitis capitata	14702	4749.37	1067.58	3.49	306.24	1481.98
Drosophila melanogaster	13152	4887.54	1078.16	3.60	299.41	1465.65
Lucilia cuprina	15622	4067.06	997.82	3.29	303.60	1343.24
EVM	17259	8021.94	1347.30	3.87	348.21	2327.30

Supplementary Table 7. Statistics on the functional annotation of *Chlorops oryzae* genes.

Database	Count	Percentage (%)
BLASTP	10763	62.36
BLASTX	10634	61.61
GO	10890	63.10
KO	6478	37.53
Map	3850	22.31
NR	14665	84.97
NT	4353	25.22
PFAM	11166	64.70
eggNOG	9214	53.39
Total_anno	14863	86.12
Total_unigene	17259	100

Notes: BLASTP: compare to Uniprot database by protein sequence. BLASTX: compare to Uniprot database by gene sequence. GO: annotate to GO database. KO: annotate to KEGG ID number. Map: annotate to KEGG pathway. NR: annotate to NR database. NT: annotate to NT database. PFAM: annotate to PFAM database. eggNOG: annotate to eggNOG database.

Supplementary Table 8. Noncoding RNA of *Chlorops oryzae*.

Type	*Chlorops oryzae*
tRNA	1378
miRNA	161
rRNA	130
snRNA	93

Supplementary Table 9. Statistics on different types of orthologous gene groups.

Species	Single-copy orthologs	Multiple-copy orthologs	Unique paralogs	Other orthologs	Unclustered genes
Zootermopsis nevadensis	2298	582	754	7203	3773
Nilaparvata lugens	1896	1602	5916	11404	15906
Bemisia tabaci	2276	701	1850	6909	2165
Nasonia vitripennis	2286	691	2964	9265	2073
Apis mellifera	2250	682	354	7346	4682
Tribolium castaneum	2273	795	752	7925	1118
Anoplophora glabripennis	2205	1006	1044	9058	1502
Lucilia cuprina	2246	761	1013	9916	1600
Chlorops oryzae	2164	989	1884	8581	3641
Ceratitis capitata	2266	727	482	9191	1496
Drosophila melanogaster	2264	728	651	8096	2216
Aedes aegypti	2027	1347	1193	9221	1996
Anopheles gambiae	2249	762	596	7952	1251
Chilo suppressalis	2213	859	904	8046	2914
Bombyx mori	2164	891	741	7835	2992

Supplementary Table 10. *Chlorops oryzae* Hi-C genomic sequencing data statistics.

Sequencer	Read length (bp)	Raw-data (bp)	Clean-data (bp)
Illumina Novaseq	2*150	61,911,682,500	57,886,033,500

Supplementary Figures:

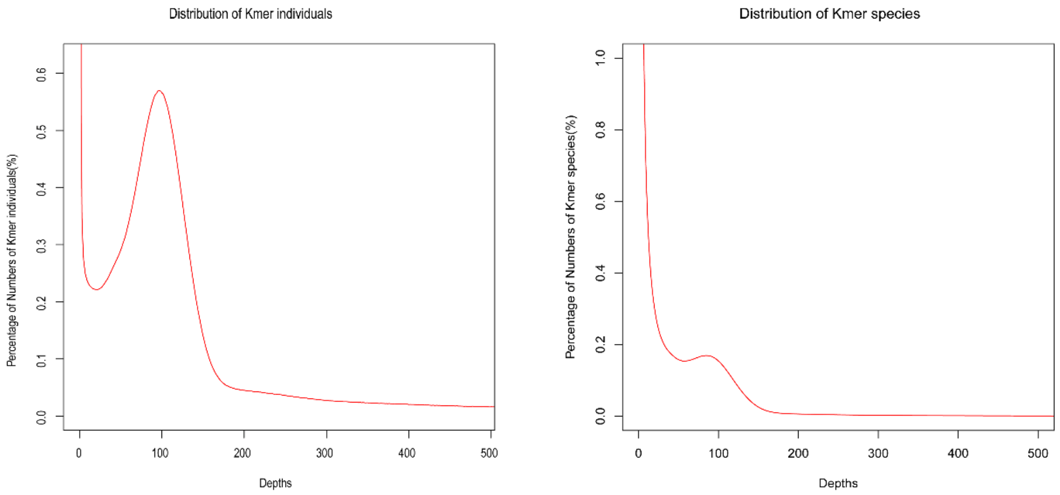

Supplementary Figure 1. K-mer frequency distribution.

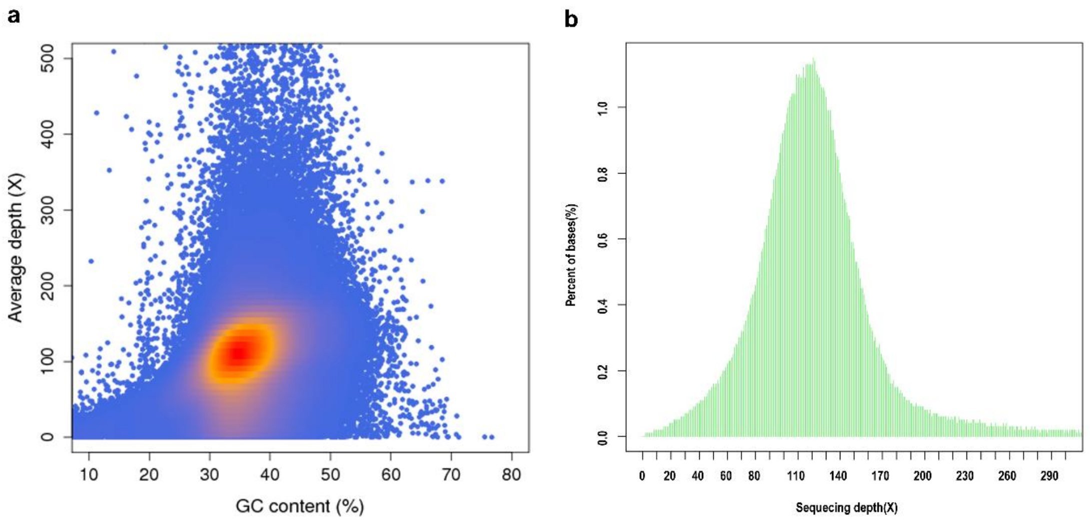

Supplementary Figure 2. G-C content of each assembled *Chlorops oryzae* gene scaffold (a) and sequencing coverage of the genome (b).

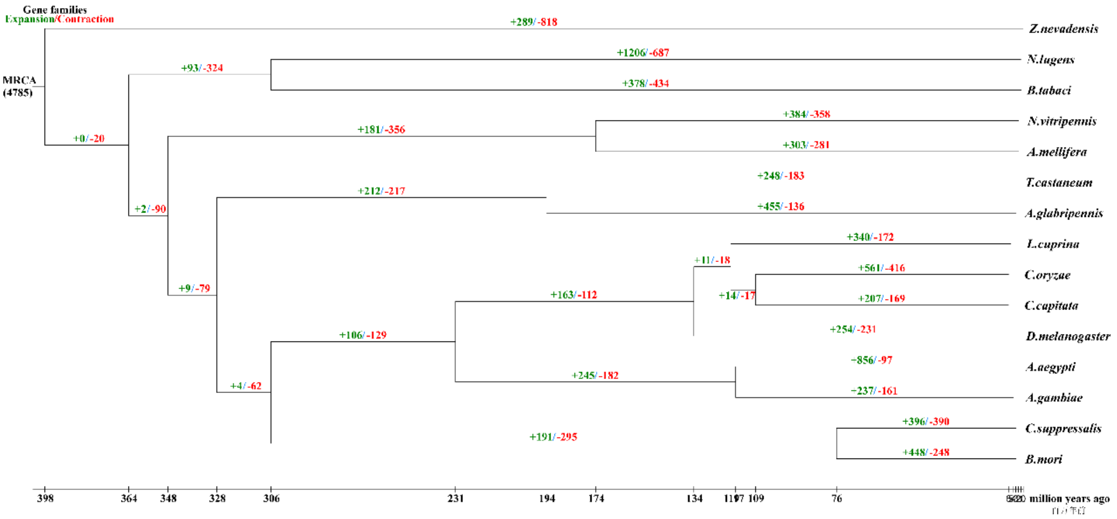

Supplementary Figure 3. Comparative genomics analysis of the *Chlorops oryzae*. The number of gene family expansion (green) and contraction (red) are shown on the branches.

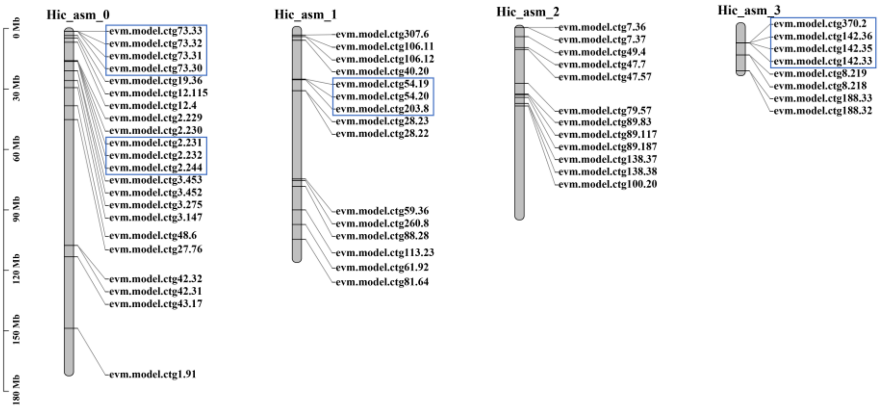

Supplementary Figure 4. The distribution of *P450* genes on the chromosome of *Chlorops oryzae*. Some genes are not mapped to the chromosome and are not shown in the figure (evm.model.ctg282.16, evm.model.ctg287.6, evm.model.ctg35.94, evm.model.ctg35.95, evm.model.ctg35.170, evm.model.ctg35.171, evm.model.ctg35.172, evm.model.ctg35.173, evm.model.ctg35.191, evm.model.ctg35.192, evm.model.ctg1501.1 and evm.model.ctg13.44). Analysis of the distribution of all 69 *P450* genes revealed 5 gene clusters with three or more *P450* genes (one cluster is not shown in the figure).

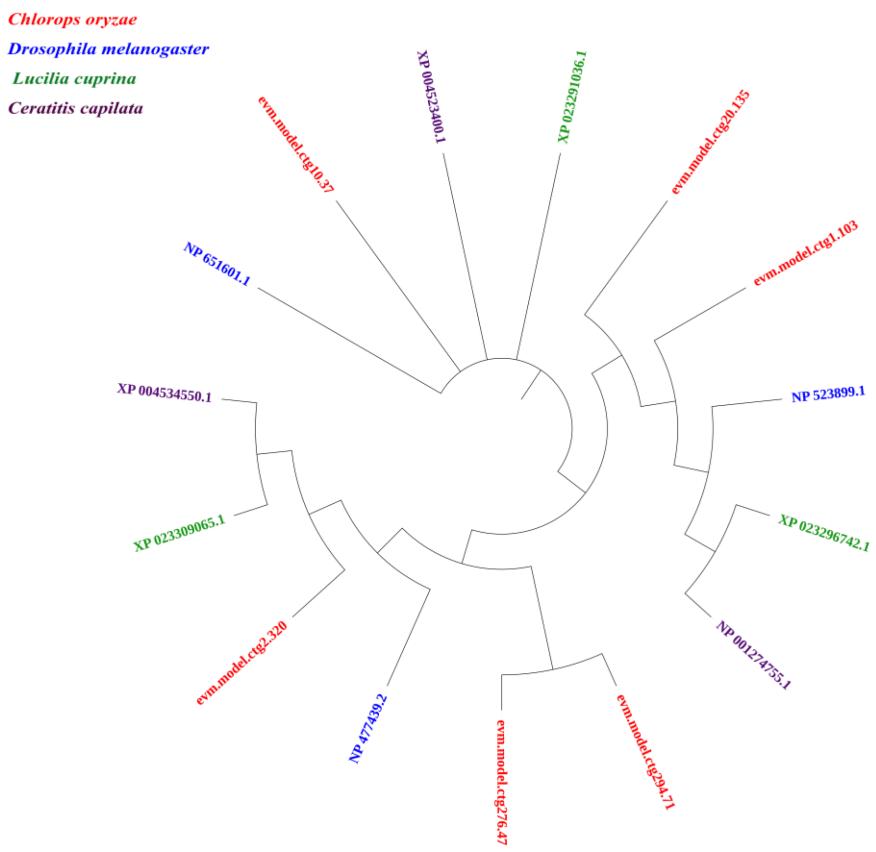

Supplementary Figure 5. Phylogenetic relationships of *HSP90* family genes from four insect species. The phylogenetic tree was constructed using the Neighbor-Joining (NJ) tree model in MEGA v.7.0. and optimized with iTOL software.

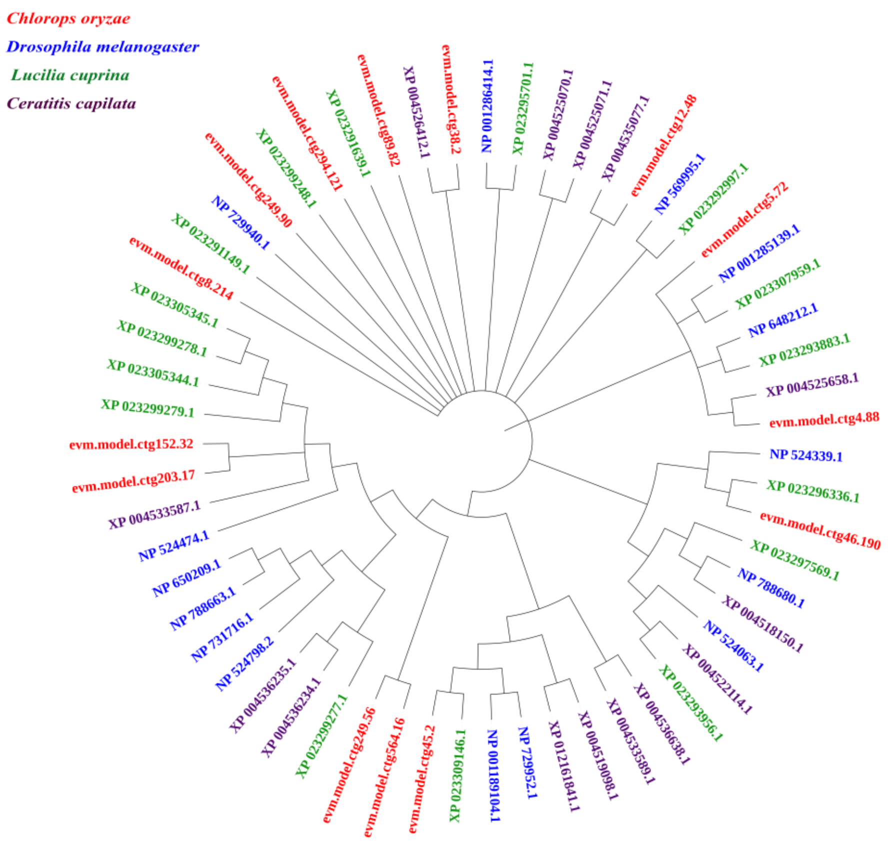

Supplementary Figure 6. Phylogenetic relationships of *HSP70* family genes from four insect species. The phylogenetic tree was constructed using the Neighbor-Joining (NJ) tree model in MEGA v.7.0. and optimized with iTOL software.

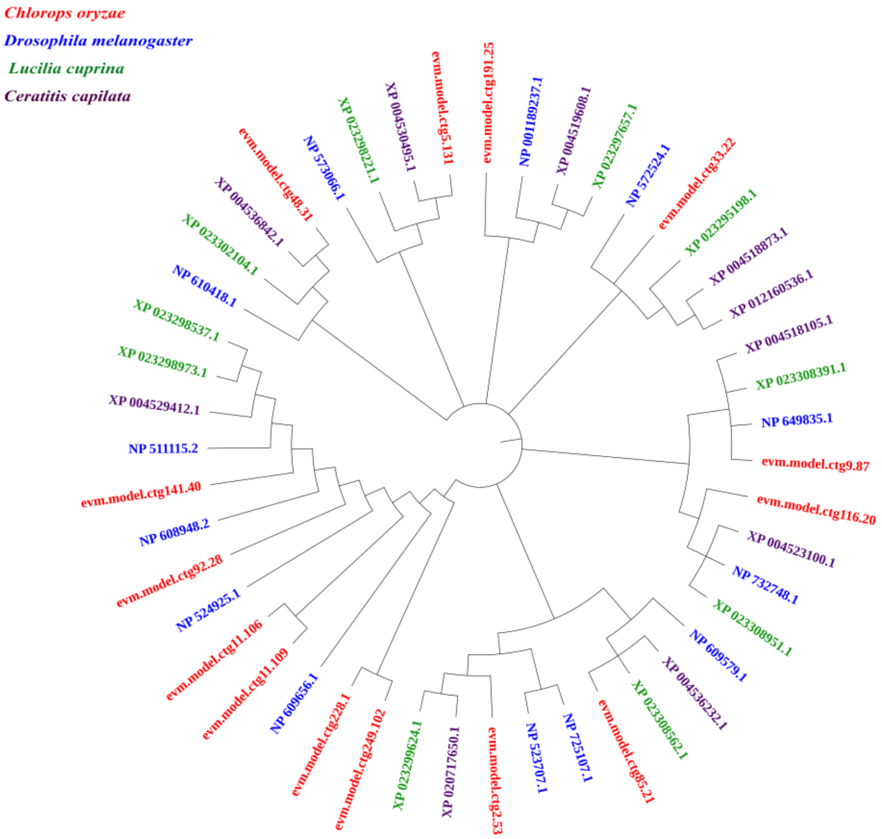

Supplementary Figure 7. Phylogenetic relationships of *HSP60* family genes from four insect species. The phylogenetic tree was constructed using the Neighbor-Joining (NJ) tree model in MEGA v.7.0. and optimized with iTOL software.

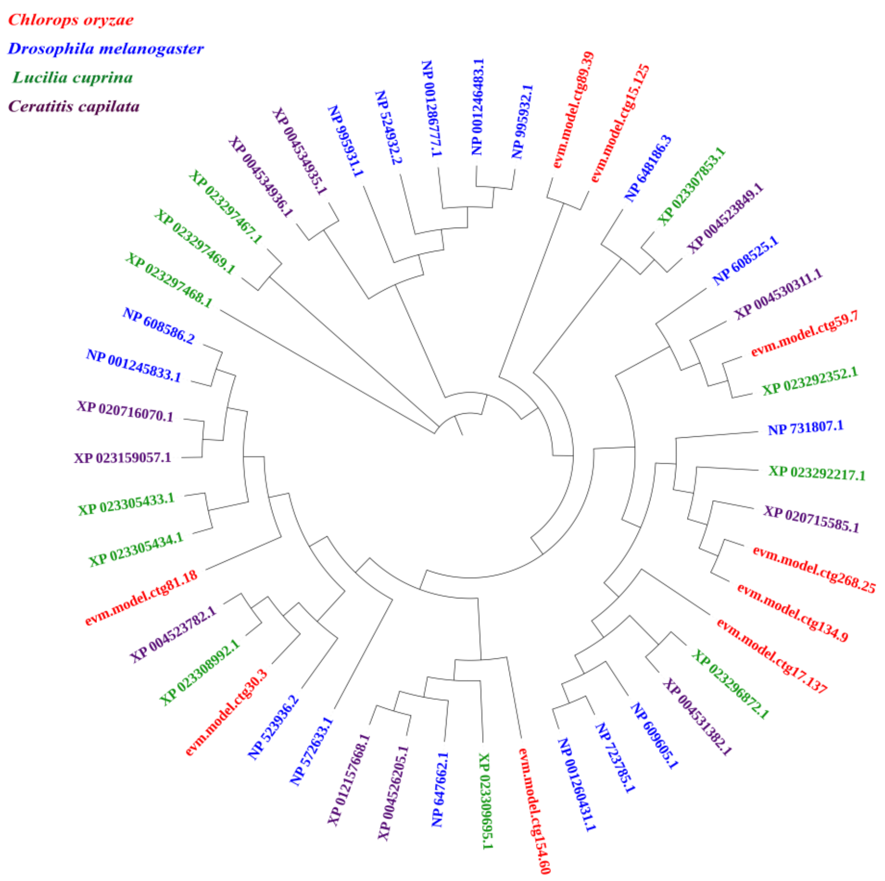

Supplementary Figure 8. Phylogenetic relationships of *HSP40* family genes from four insect species. The phylogenetic tree was constructed using the Neighbor-Joining (NJ) tree model in MEGA v.7.0. and optimized with iTOL software.

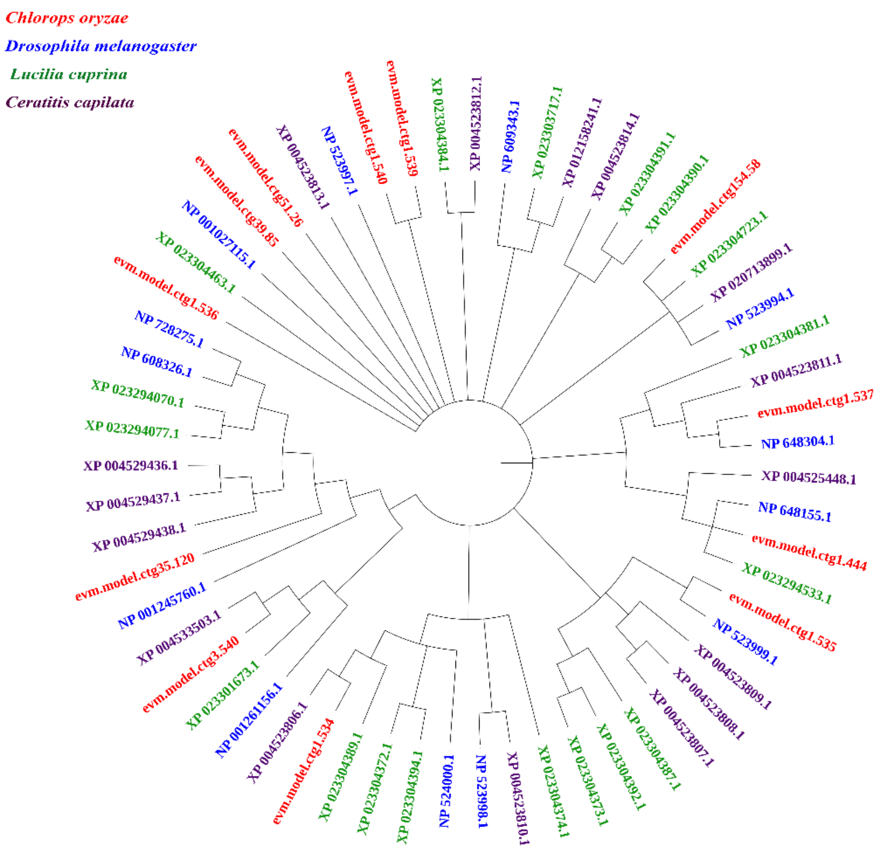

Supplementary Figure 9. Phylogenetic relationships of *sHSP* family genes from four insect species. The phylogenetic tree was constructed using the Neighbor-Joining (NJ) tree model in MEGA v.7.0. and optimized with iTOL software.

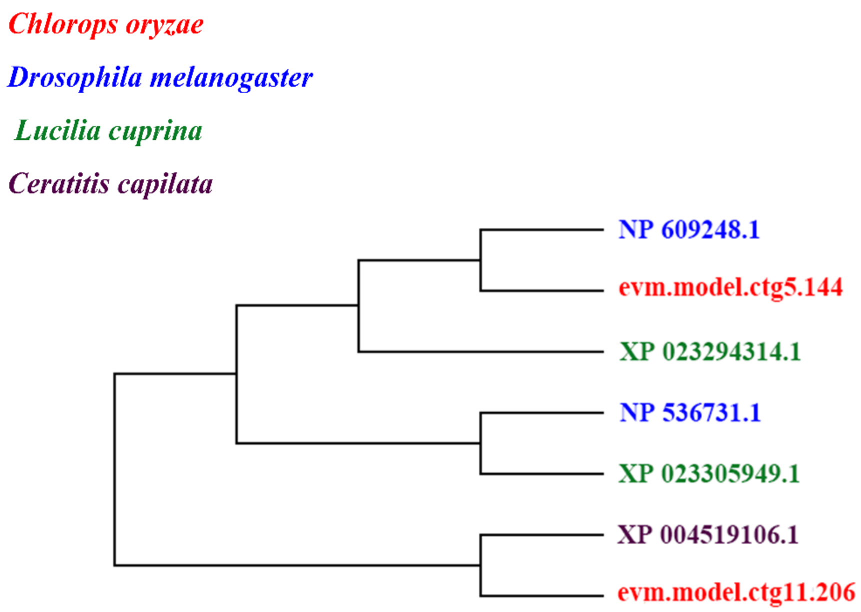

Supplementary Figure 10. Phylogenetic tree of insect *catalase* (*CAT*) genes. The tree was constructed using the Neighbor-Joining (NJ) method in MEGA v.7.0. and optimized with iTOL software.

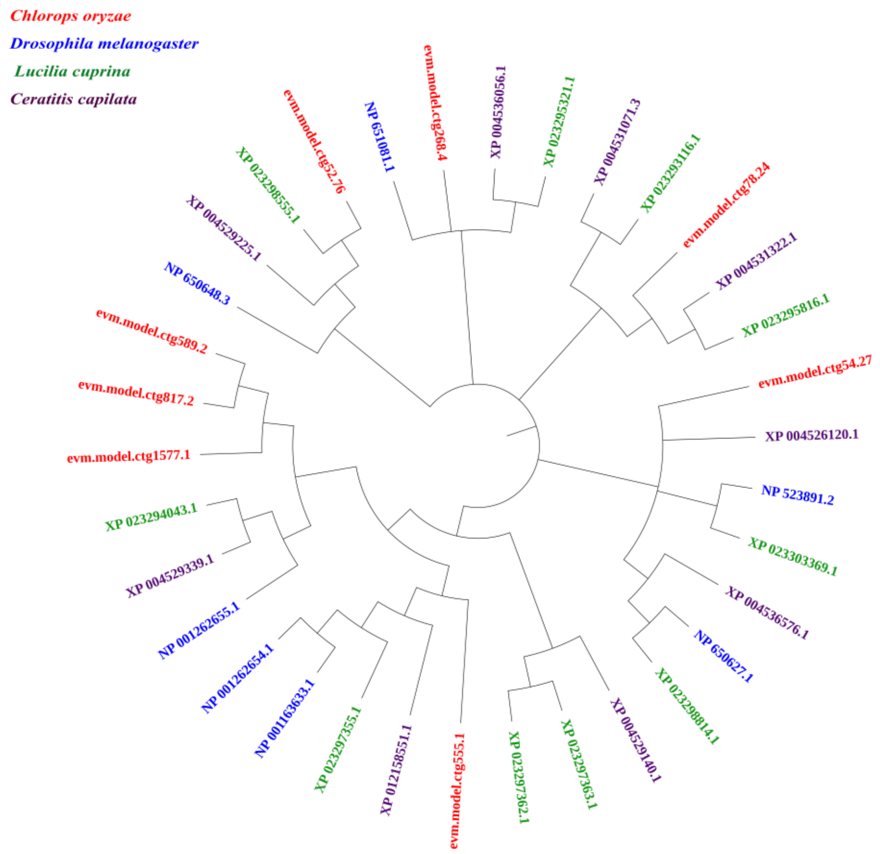

Supplementary Figure 11. Phylogenetic tree of insect *peroxidase* (*POD*) genes. The tree was constructed using the Neighbor-Joining (NJ) method in MEGA v.7.0. and optimized with iTOL software.

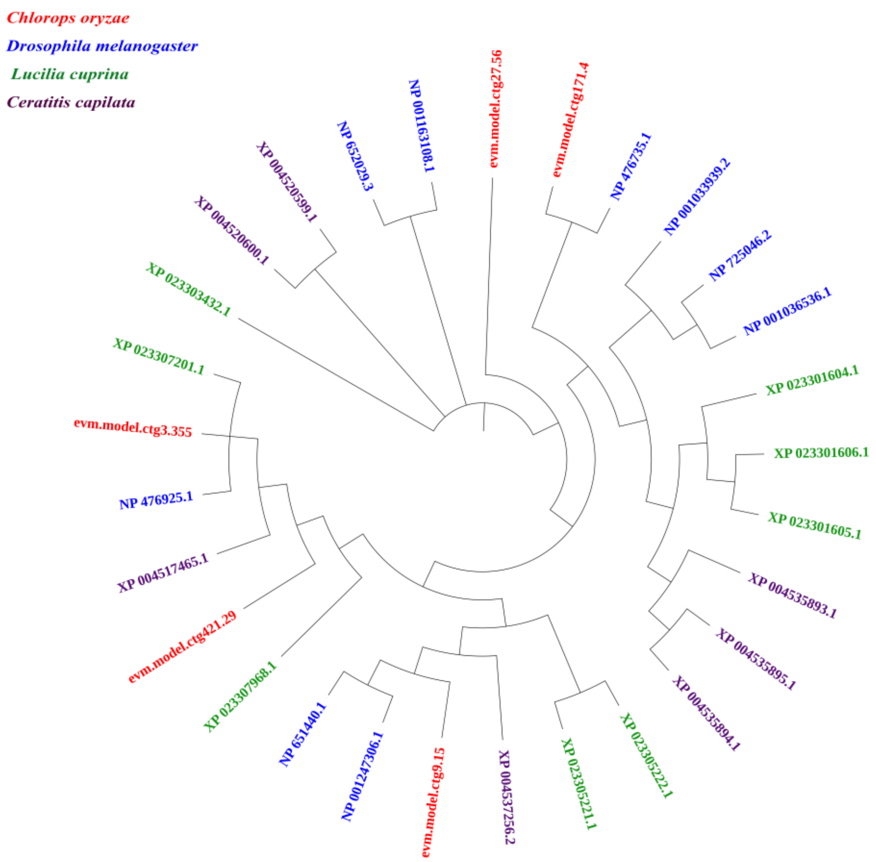

Supplementary Figure 12. Phylogenetic tree of insect *superoxide dismutase* (*SOD*) genes. The tree was constructed using the Neighbor-Joining (NJ) method in MEGA v.7.0. and optimized with iTOL software.

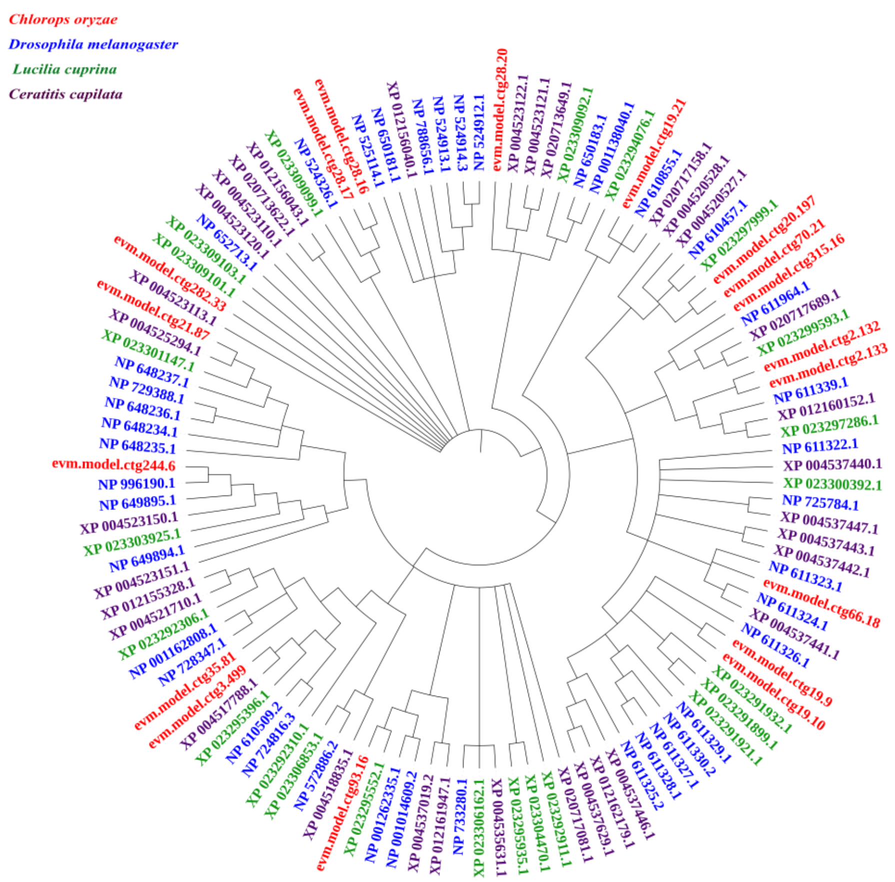

Supplementary Figure 13. Phylogenetic tree of insect *glutathione-S-transferase*'s (*GST*) genes.

The tree was constructed using the Neighbor-Joining (NJ) method in MEGA v.7.0. and optimized with iTOL software.

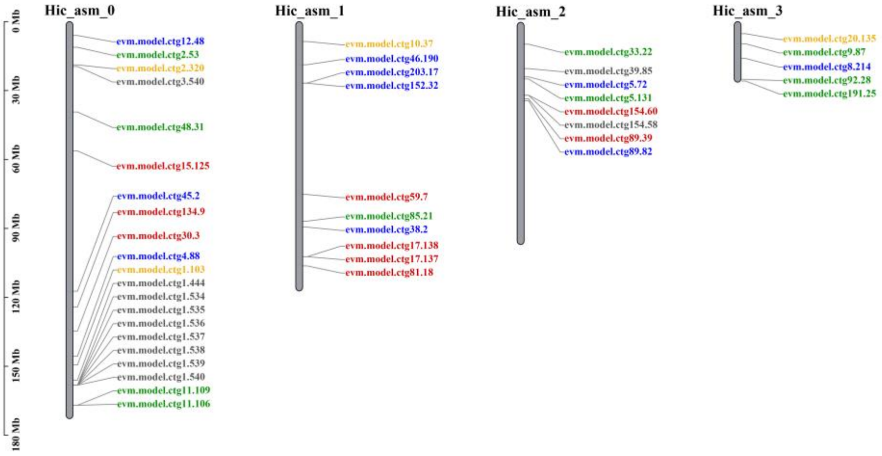

Supplementary Figure 14. Distribution of *HSP* genes on the chromosome of *Chlorops oryzae*. Some genes are not mapped to the chromosome and are not shown in the figure (evm.model.ctg276.47-90, evm.model.ctg294.71-90, evm.model.ctg294.121-70, evm.model.ctg268.25-40, evm.model.ctg249.56-70, evm.model.ctg249.90-70, evm.model.ctg249.102-60, evm.model.ctg51.26-20, evm.model.ctg228.1-60, evm.model.ctg35.120-20, evm.model.ctg141.40-60, evm.model.ctg564.16-70 and evm.model.ctg116.20-60). Names of different genes are shown in different colors; *HSP20* genes in gray, *HSP40* genes in red, *HSP60* genes in green, *HSP70* genes in blue and *HSP90* genes in gold.

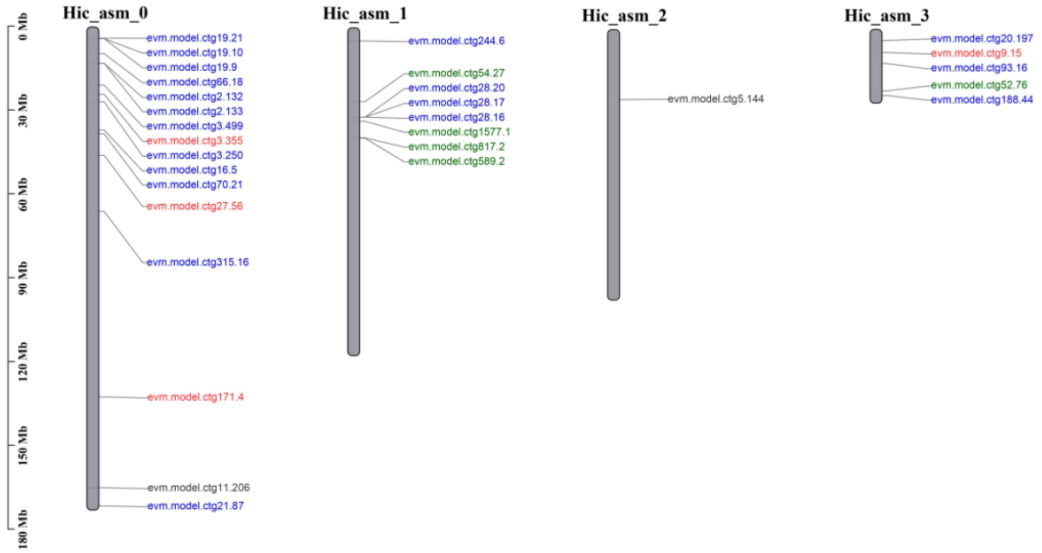

Supplementary Figure 15. The distribution of antioxidant genes on the chromosome of *Chlorops oryzae*. Some genes are not mapped to the chromosome and are not shown in the figure (evm.model.ctg78.24-POD, evm.model.ctg555.1-POD, evm.model.ctg35.81-GST, evm.model.ctg35.180-GST, evm.model.ctg282.33-GST, evm.model.ctg268.4-POD and evm.model.ctg421.29-SOD). Names of different gene families are shown in different colors; *CAT* genes in gray, *POD* genes in green, *SOD* genes in red and *GST* genes in blue.

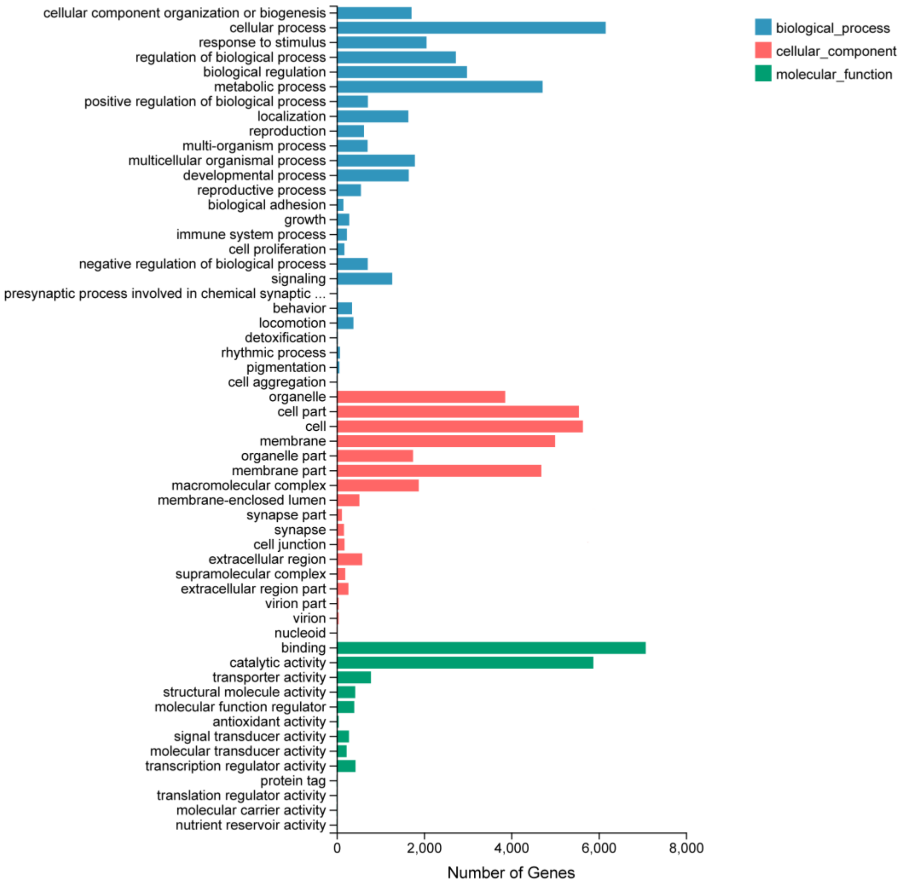

Supplementary Figure 16. Gene ontology classification of differentially expressed *Chlorops oryzae* unigenes.

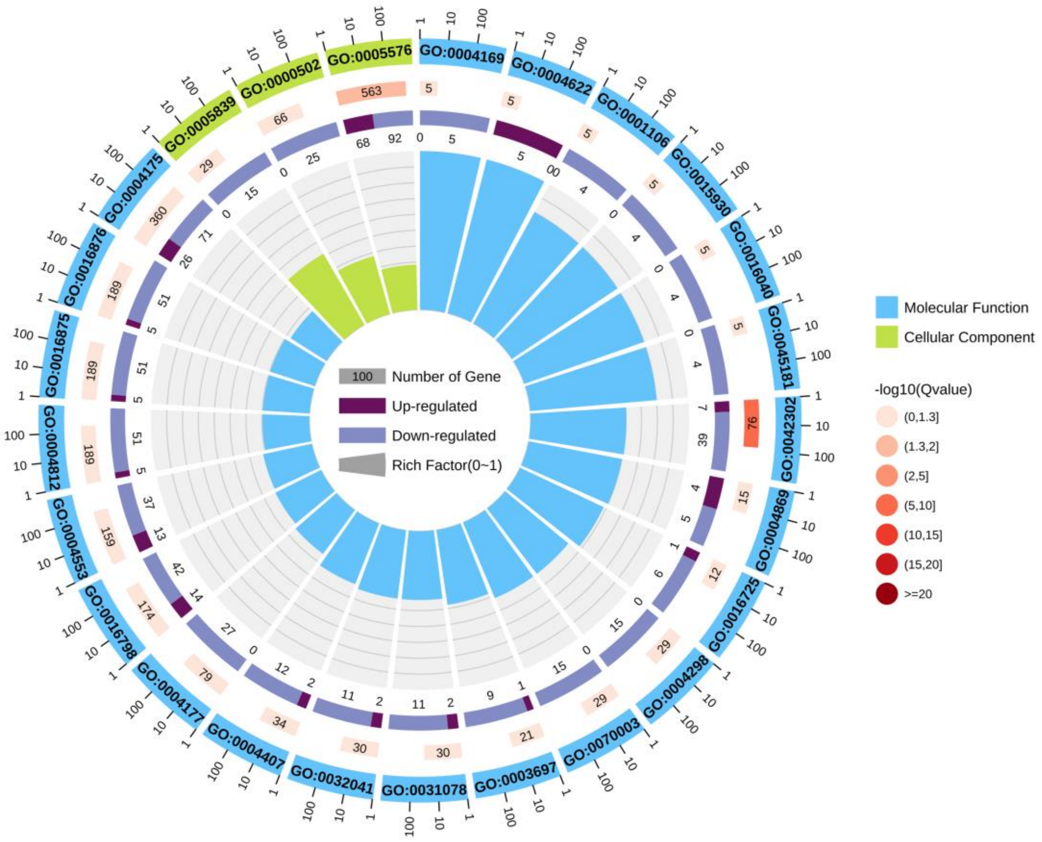

Supplementary Figure 17. GO term analysis of upregulated pathwys in 33 ℃ vs 39 ℃ was performed. The first ring indicates top 20 GO term and the number of the genes corresponds to the outer ring. The second ring indicates the number of the genes in the transcriptome background and -log10(Q value) for enrichment of the upregulated genes for the specified biological process. The third ring indicates the ratio of the upregulated genes (deep purple) and downregulated genes (light purple). The fourth ring indicates the enrichment factor of each GO term.

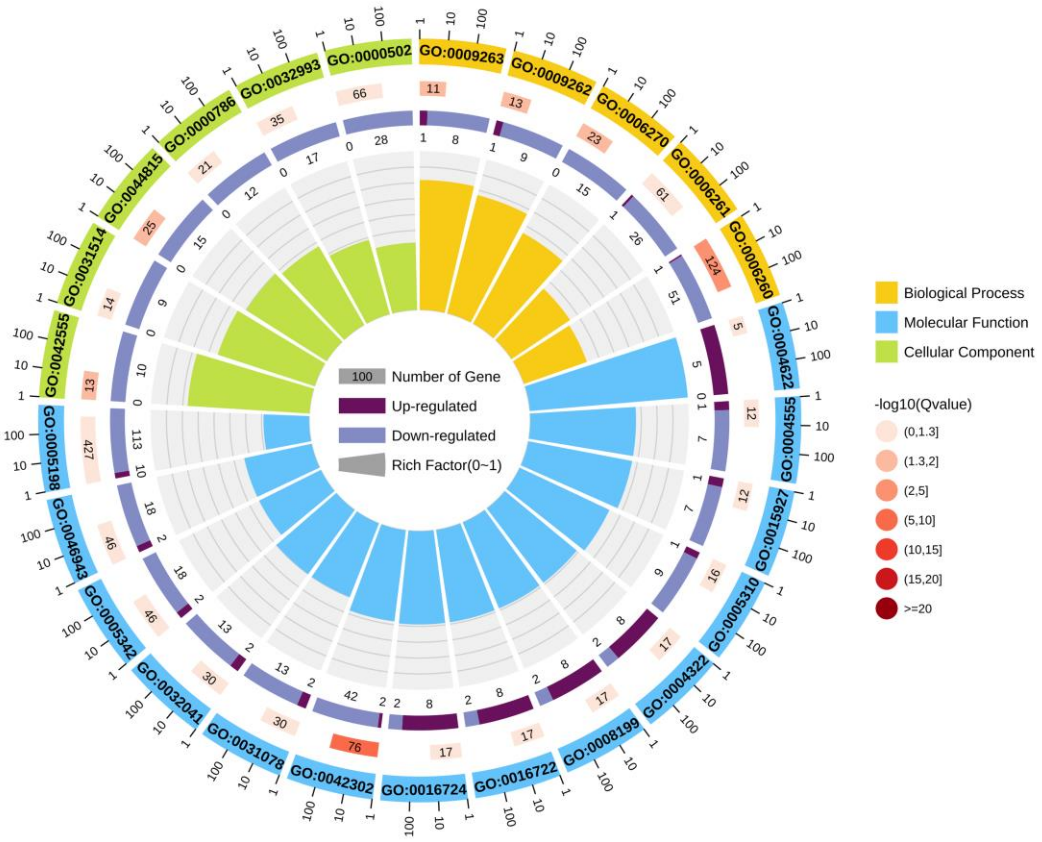

Supplementary Figure 18. GO term analysis of upregulated pathwys in 24 ℃ vs 39 ℃ was performed. The first ring indicates top 20 GO term and the number of the genes corresponds to the outer ring. The second ring indicates the number of the genes in the transcriptome background and -log10(Q value) for enrichment of the upregulated genes for the specified biological process. The third ring indicates the ratio of the upregulated genes (deep purple) and downregulated genes (light purple). The fourth ring indicates the enrichment factor of each GO term.

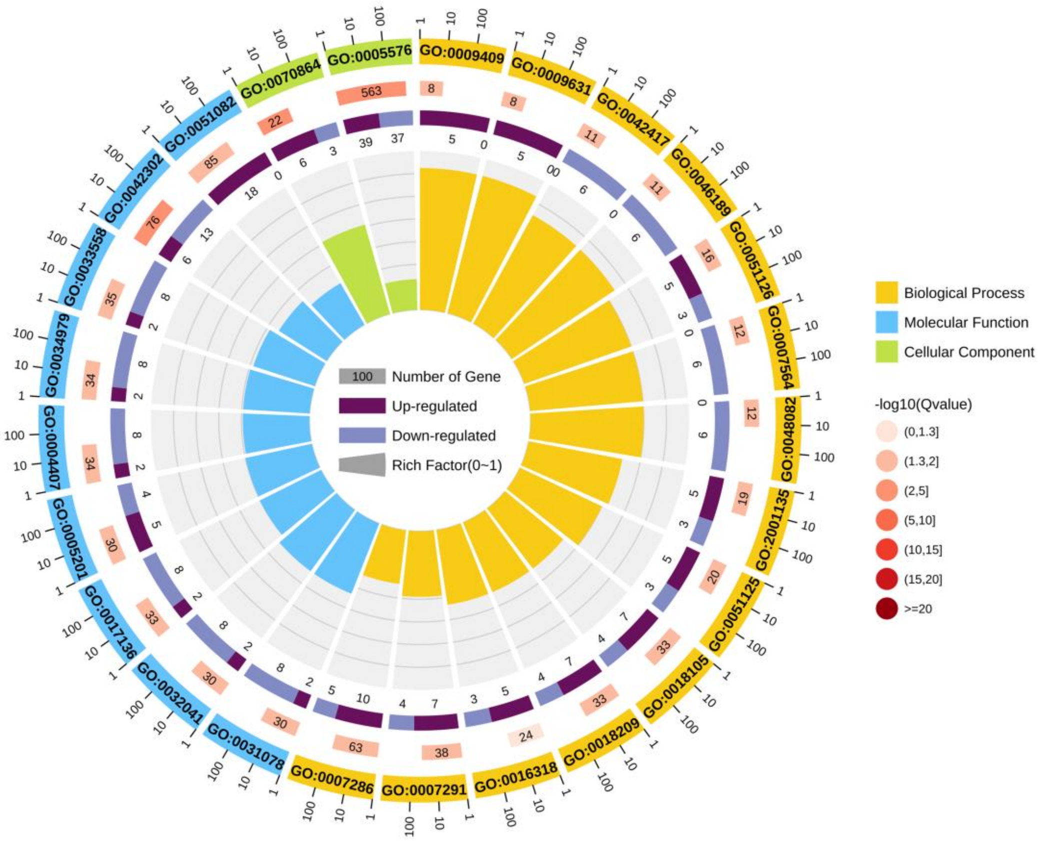

Supplementary Figure 19. GO term analysis of upregulated pathwys in 24 ℃ vs 33 ℃ was performed. The first ring indicates top 20 GO term and the number of the genes corresponds to the outer ring. The second ring indicates the number of the genes in the transcriptome background and -log10(Q value) for enrichment of the upregulated genes for the specified biological process. The third ring indicates the ratio of the upregulated genes (deep purple) and downregulated genes (light purple). The fourth ring indicates the enrichment factor of each GO term.

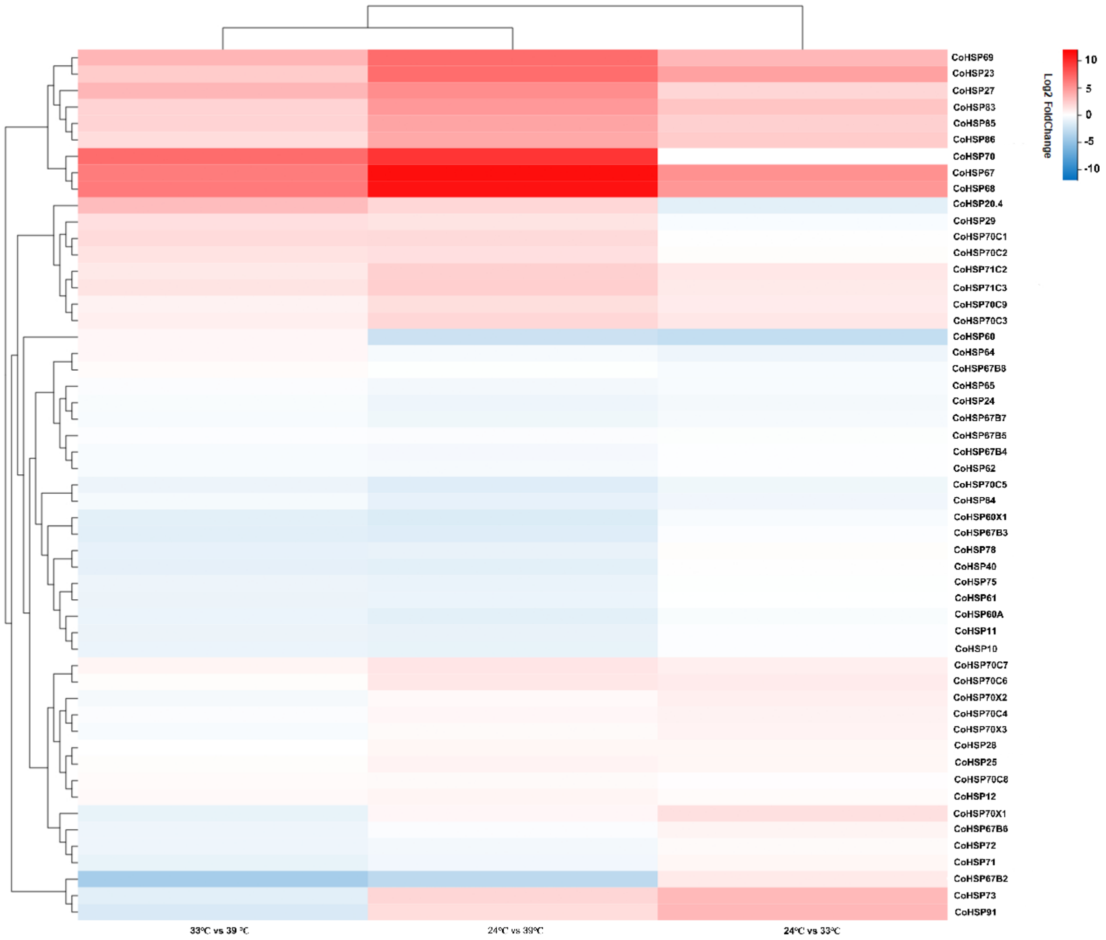

Supplementary Figure 20. Hierarchical cluster analysis of *heat shock proteins* (*HSPs*) genes of *Chlorops oryzae* larvae subject to temperature stress.

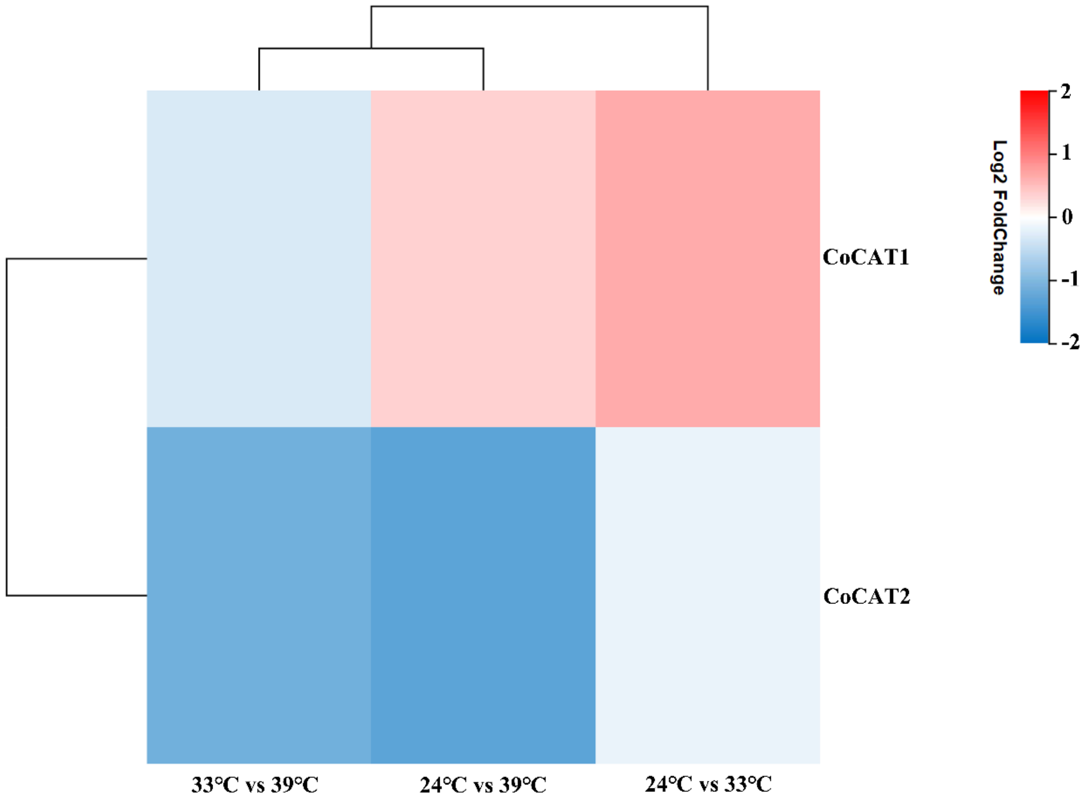

Supplementary Figure 21. Hierarchical cluster analysis of *catalase* (*CAT*) genes of *Chlorops oryzae* larvae subject to temperature stress.

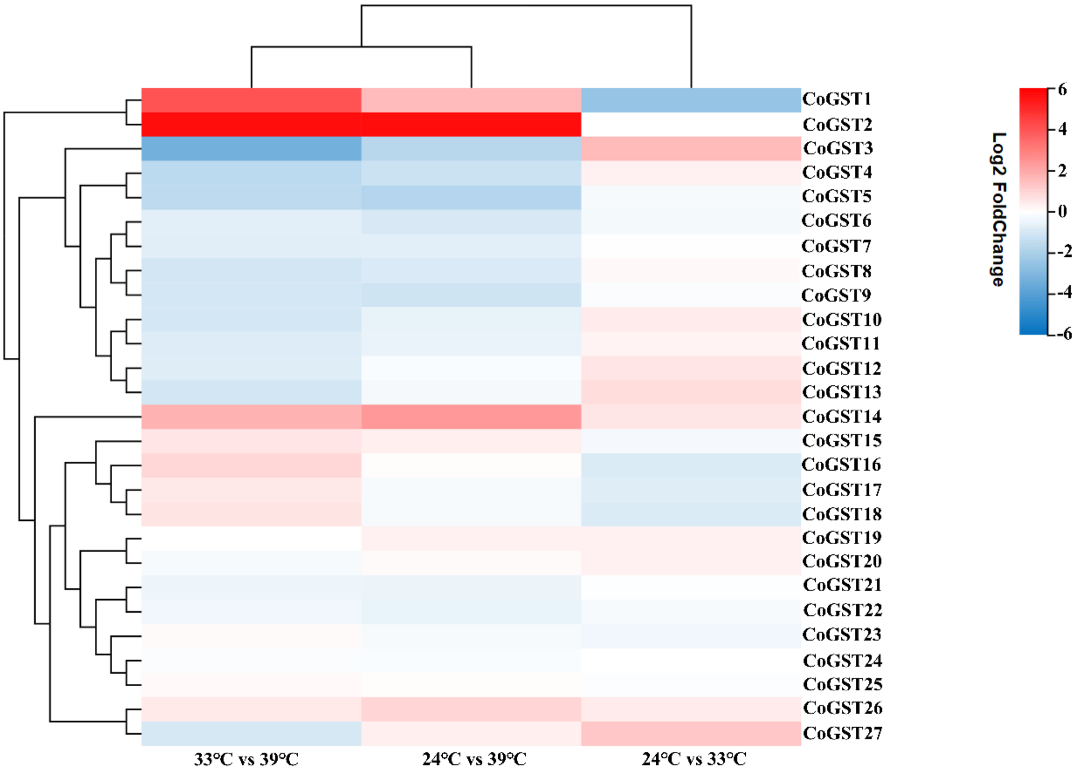

Supplementary Figure 22. Hierarchical cluster analysis of *glutathione S transferase* (*GST*) genes of *Chlorops oryzae* larvae subject to temperature stress.

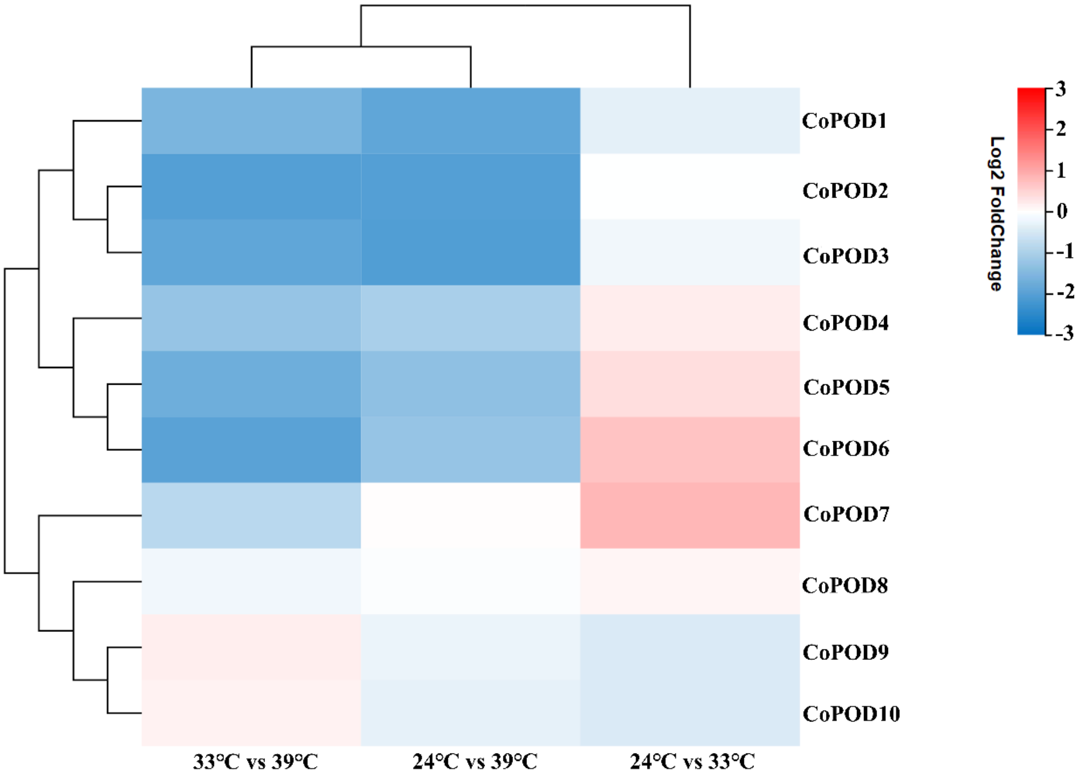

Supplementary Figure 23. Hierarchical cluster analysis of *peroxidase* (*POD*) genes of *Chlorops oryzae* larvae subject to temperature stress.

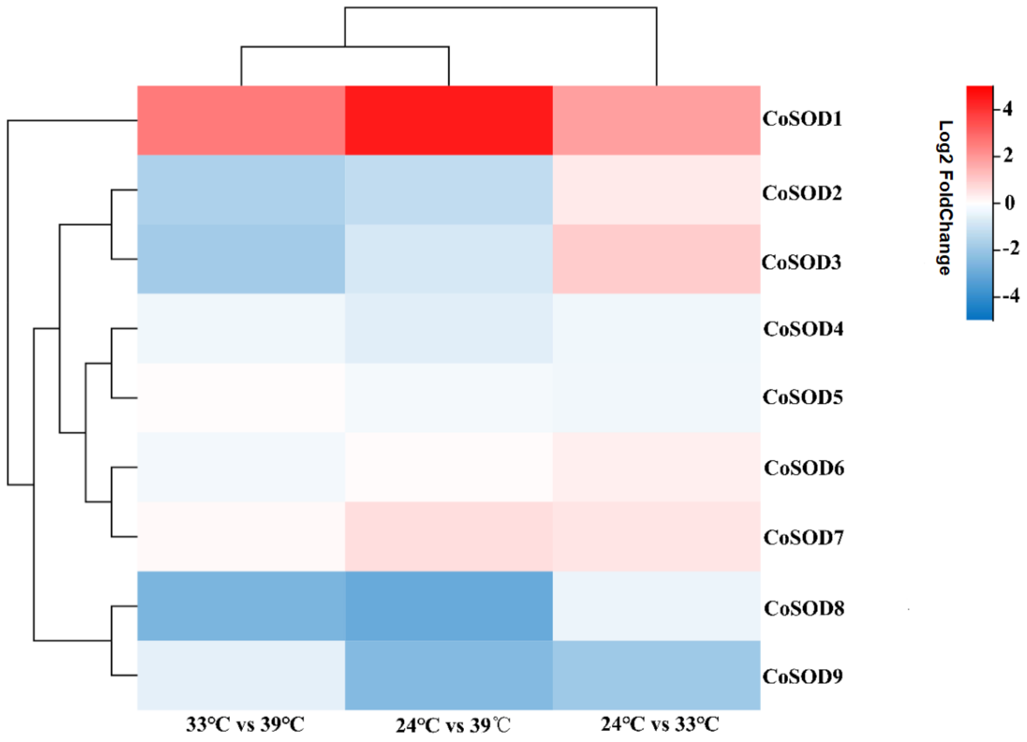

Supplementary Figure 24. Hierarchical cluster analysis of *superoxidase dismutase* (*SOD*) genes of *Chlorops oryzae* larvae subject to temperature stress.

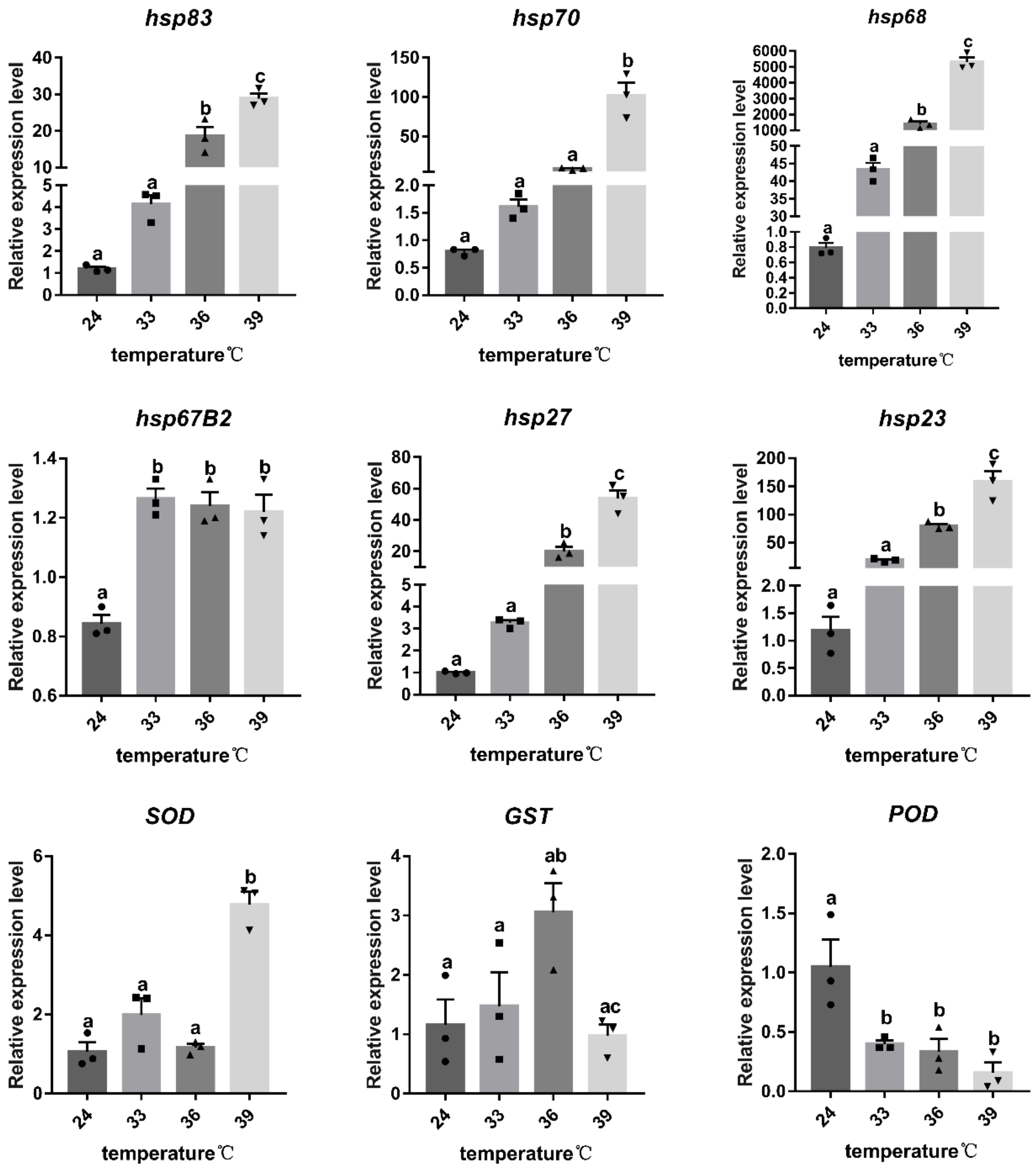

Supplementary Figure 25. Expression profiles of stress and antioxidant genes in *Chlorops oryzae* larvae subject to temperature stress. Each treatment had 3 biological replicates. Gene expression was normalized to that of the *RPS15* and *RP49* genes. For each gene, same letters above the bars indicate no significant differences, different letters above the bars indicate significant differences, $P<0.05$ (ANOVA). Error bars are SEM.

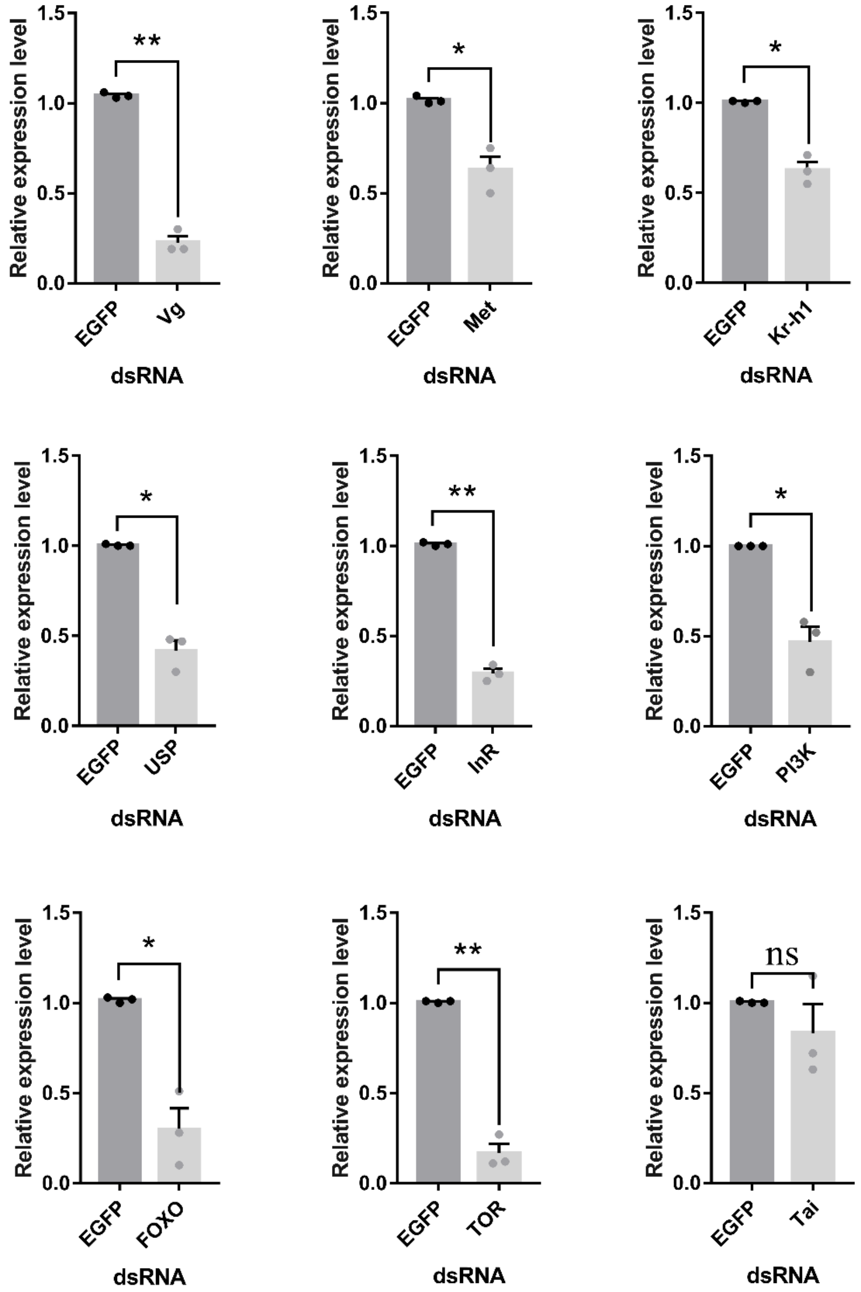

Supplementary Figure 26. mRNA levels determined by qRT-PCR. Each gene had 3 biological replicates. Data were calculated using the $2^{-\Delta\Delta Ct}$ method normalized to the expression of *RPS15* and *RP49*. Asterisks indicate significant differences (Student's *t* test: *P < 0.05, **P < 0.01); ns: not significant. Error bars are SEM.

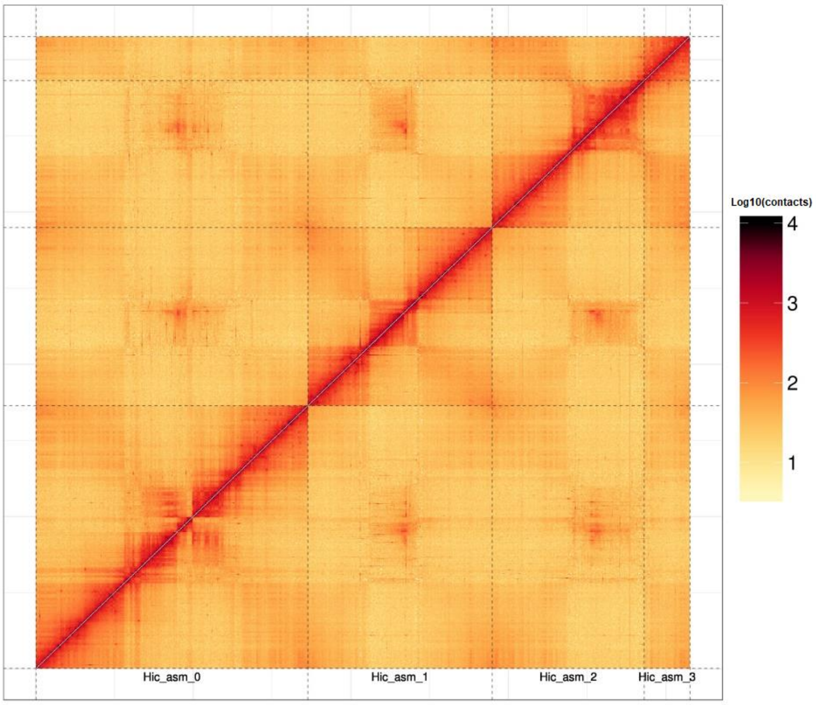

Supplementary Figure 27. Genome-wide, all-by-all Hi-C interaction in *Chlorops oryzae*.
