# Supplementary material for: A chromosome-level genome assembly provides insights into the environmental adaptability and outbreaks of Chlorops oryzae
Source: Commun Biol. 2022 Aug 26;5:881. doi: 10.1038/s42003-022-03850-7 (PMC9418232; doi:10.1038/s42003-022-03850-7)
Supplement: Supplementary file 2 — Description of Additional Supplementary Files [file 42003_2022_3850_MOESM2_ESM.docx]

**Description of Additional Supplementary Files**

**File name:** Supplementary Data 1
**Description:** Summary of different gene families in Chlorops oryzae.

**File name:** Supplementary Data 2 **Description:** DET total with fpkm.

**File name:** Supplementary Data 3 **Description:** Protein sequences of P450s used to construct phylogenetic tree.

**File name:** Supplementary Data 4 **Description:** Primers used in this study.

**File name:** Supplementary Data 5 **Description:** Hi-C data of Chlorops oryzae.
