# Supplementary material for: A chromosome-level genome assembly provides insights into the environmental adaptability and outbreaks of Chlorops oryzae
Source: Commun Biol. 2022 Aug 26;5:881. doi: 10.1038/s42003-022-03850-7 (PMC9418232; doi:10.1038/s42003-022-03850-7)
Supplement: Supplementary file 3 — Supplementary Data 1 [file 42003_2022_3850_MOESM3_ESM.docx]

**Supplementary Data 1. Summary of different gene families in *Chlorops oryzae*.**

| Gene family | Gene ID | Annotation | Gene family | Gene ID | Annotation |
| --- | --- | --- | --- | --- | --- |
| HSP90 | evm.model.ctg1.103 | heat shock protein 83 | HSP40 | evm.model.ctg30.3 | dnaJ homolog 1 |
|  | evm.model.ctg20.135 | heat shock protein 90 |  | evm.model.ctg81.18 | dnaJ homolog1 X1 |
|  | evm.model.ctg10.37 | heat shock protein 90 -like |  | evm.model.ctg268.25 | dnaJ homolog subfamily A member 4 |
|  | evm.model.ctg294.71 | heat shock protein 87 |  | evm.model.ctg59.7 | dnaJ homolog shv |
|  | evm.model.ctg276.47 | heat shock protein 90 -like |  | evm.model.ctg134.9 | dnaJ homolog subfamily A |
|  | evm.model.ctg2.320 | heat shock protein 75 kDa |  | evm.model.ctg89.39 | dnaJ homolog 2 |
| HSP70 | evm.model.ctg249.90 | molecular chaperone DnaK |  | evm.model.ctg154.60 | dnaJ homolog subfamily B member 13 |
|  | evm.model.ctg38.2 | heat shock 70 kDa protein cognate 5 |  | evm.model.ctg15.125 | dnaJ homolog 3 |
|  | evm.model.ctg294.121 | molecular chaperone DnaK-like |  | evm.model.ctg17.137 | dnaJ homolog subfamily A member 2 |
|  | evm.model.ctg5.72 | heat shock 70 kDa protein cognate 3 |  | evm.model.ctg17.138 | dnaJ homolog subfamily A member 4 |
|  | evm.model.ctg203.17 | heat shock protein 68 | CAT | evm.model.ctg11.206 | catalase |
|  | evm.model.ctg152.32 | heat shock protein 70 |  | evm.model.ctg5.144 | catalase |
|  | evm.model.ctg12.48 | heat shock 70 kDa protein cognate 6 | P450 | evm.model.ctg61.92 | cytochrome P450 4d1 isoform X2 |
|  | evm.model.ctg46.190 | heat shock 70 kDa protein cognate 2 |  | evm.model.ctg100.20 | cytochrome P450 4ae1 isoform X1 |
|  | evm.model.ctg8.214 | heat shock 70 kDa protein cognate 4 |  | evm.model.ctg2.232 | cytochrome P450 6a21 |
|  | evm.model.ctg45.2 | heat shock 70 kDa protein 4 isoform X2 |  | evm.model.ctg7.36 | cytochrome P450 18a1 |
|  | evm.model.ctg564.16 | Hsp70 family protein |  | evm.model.ctg307.6 | cytochrome P450 4c3 |
|  | evm.model.ctg249.56 | Hsp70 family protein |  | evm.model.ctg282.16 | cytochrome P450 4d14 |
|  | evm.model.ctg89.82 | Hsp70-like |  | evm.model.ctg106.11 | cytochrome P450 6a2 |
|  | evm.model.ctg4.88 | heat shock 70 kDa protein 14 |  | evm.model.ctg260.8 | cytochrome P450 4ac1 |
| HSP60 | evm.model.ctg5.131 | T-complex protein 1 subunit zeta |  | evm.model.ctg40.20 | cytochrome P450 12e1 |
|  | evm.model.ctg116.20 | T-complex protein 1 subunit alpha |  | evm.model.ctg2.231 | cytochrome P450 6a23 |
|  | evm.model.ctg85.21 | T-complex protein 1 subunit delta |  | evm.model.ctg1501.1 | cytochrome P450 4g1-like |
|  | evm.model.ctg2.53 | T-complex protein 1 subunit epsilon |  | evm.model.ctg113.23 | cytochrome P450 313a4 |
|  | evm.model.ctg191.25 | T-complex protein 1 subunit gamma |  | evm.model.ctg35.94 | cytochrome P450 4g1 |
|  | evm.model.ctg48.31 | T-complex protein 1 subunit theta |  | evm.model.ctg3.275 | cytochrome P450 6a2-like |
|  | evm.model.ctg33.22 | T-complex protein 1 subunit beta |  | evm.model.ctg2.229 | cytochrome P450 6a9 |
|  | evm.model.ctg9.87 | T-complex protein 1 subunit eta |  | evm.model.ctg35.95 | cytochrome P450 4g2 |
|  | evm.model.ctg249.102 | chaperonin GroEL |  | evm.model.ctg2.230 | cytochrome P450 6a13 |
|  | evm.model.ctg228.1 | chaperonin GroEL |  | evm.model.ctg28.23 | cytochrome P450 9f2 |
|  | evm.model.ctg141.40 | heat shock protein 60A isoform X2 |  | evm.model.ctg89.117 | cytochrome P450 305a1 |
|  | evm.model.ctg92.28 | heat shock protein 60A |  | evm.model.ctg79.57 | cytochrome P450 311a1 |
|  | evm.model.ctg11.109 | heat shock protein 60A1 |  | evm.model.ctg188.32 | cytochrome P450 6d5 |
|  | evm.model.ctg11.106 | heat shock protein 60 |  | evm.model.ctg73.31 | cytochrome P450 6a14 |
| sHSP | evm.model.ctg1.538 | hsp23 |  | evm.model.ctg3.147 | cytochrome P450 6g1 |
|  | evm.model.ctg1.540 | hsp23-like |  | evm.model.ctg47.7 | cytochrome P450 303a1 |
|  | evm.model.ctg1.539 | hsp23-like |  | evm.model.ctg2.244 | cytochrome P450 4e2 |
|  | evm.model.ctg1.534 | hsp27-like |  | evm.model.ctg73.33 | cytochrome P450 6a15 |
|  | evm.model.ctg1.535 | hsp23-like |  | evm.model.ctg12.115 | cytochrome P450 4p1 |
|  | evm.model.ctg39.85 | hsp23-like |  | evm.model.ctg59.36 | cytochrome P450 6d3 |
|  | evm.model.ctg3.540 | hsp21.3 |  | evm.model.ctg35.173 | cytochrome P450 28d1 |
|  | evm.model.ctg1.536 | hsp27-like |  | evm.model.ctg8.218 | cytochrome P450 313a5 |
|  | evm.model.ctg51.26 | hsp25 |  | evm.model.ctg188.33 | cytochrome P450 6d6 |
|  | evm.model.ctg1.537 | hsp27-like |  | evm.model.ctg203.8 | cytochrome P450 304a1 |
|  | evm.model.ctg154.58 | hsp20 |  | evm.model.ctg138.38 | cytochrome P450 4d1 |
|  | evm.model.ctg35.120 | hsp12.2 |  | evm.model.ctg3.453 | cytochrome P450 6g2 |
|  | evm.model.ctg1.444 | hsp27 |  | evm.model.ctg54.19 | cytochrome P450 12a2 |
| POD | evm.model.ctg555.1 | peroxidase |  | evm.model.ctg73.32 | cytochrome P450 6a8 |
|  | evm.model.ctg817.2 | peroxidase |  | evm.model.ctg48.6 | cytochrome P450 6u1 |
|  | evm.model.ctg589.2 | peroxidase |  | evm.model.ctg89.187 | cytochrome P450 6v1 |
|  | evm.model.ctg52.76 | chorion peroxidase |  | evm.model.ctg47.57 | cytochrome P450 4g15 |
|  | evm.model.ctg54.27 | peroxidase |  | evm.model.ctg142.33 | cytochrome P450 313a6 |
|  | evm.model.ctg78.24 | heme peroxidase 2 isoform X2 |  | evm.model.ctg73.30 | cytochrome P450 317a1 |
|  | evm.model.ctg268.4 | chorion peroxidase-like |  | evm.model.ctg43.17 | cytochrome P450 302a1 |
|  | evm.model.ctg1577.1 | peroxidase isoform X2 |  | evm.model.ctg12.4 | cytochrome P450 28d2 |
| SOD | evm.model.ctg171.4 | superoxide dismutase [Cu-Zn] |  | evm.model.ctg42.32 | cytochrome P450 9b2 |
|  | evm.model.ctg27.56 | superoxide dismutase |  | evm.model.ctg3.452 | cytochrome P450 6t3 |
|  | evm.model.ctg9.15 | superoxide dismutase [Cu-Zn] isoform X2 |  | evm.model.ctg81.64 | cytochrome P450 6d1 |
|  | evm.model.ctg3.355 | superoxide dismutase [Mn] |  | evm.model.ctg19.36 | cytochrome P450 4aa1 |
|  | evm.model.ctg421.29 | superoxide dismutase |  | evm.model.ctg28.22 | cytochrome P450 9f3 |
| GST | evm.model.ctg20.197 | glutathione S-transferase 1 |  | evm.model.ctg35.192 | cytochrome P450 28d3 |
|  | evm.model.ctg188.44 | glutathione S-transferase |  | evm.model.ctg287.6 | cytochrome P450 307a1 |
|  | evm.model.ctg70.21 | glutathione S-transferase 1-like |  | evm.model.ctg42.31 | cytochrome P450 9b3 |
|  | evm.model.ctg28.17 | glutathione S-transferase D1 |  | evm.model.ctg35.171 | cytochrome P450 28a5 |
|  | evm.model.ctg28.16 | glutathione S-transferase 1-1 |  | evm.model.ctg27.76 | cytochrome P450 301a1 |
|  | evm.model.ctg19.21 | glutathione S-transferase E14 |  | evm.model.ctg89.83 | cytochrome P450 4d2 |
|  | evm.model.ctg35.81 | glutathione S-transferase theta-1 |  | evm.model.ctg7.37 | cytochrome P450 306a1 |
|  | evm.model.ctg2.132 | glutathione S-transferase 1-like |  | evm.model.ctg13.44 | cytochrome P450 315a1 |
|  | evm.model.ctg2.133 | glutathione S-transferase 1-like |  | evm.model.ctg88.28 | cytochrome P450 309a2 |
|  | evm.model.ctg21.87 | glutathione S-transferase omega 1 |  | evm.model.ctg35.191 | cytochrome P450 309a1 |
|  | evm.model.ctg16.5 | glutathione S-transferase S1 |  | evm.model.ctg49.4 | cytochrome P450 318a1 |
|  | evm.model.ctg19.9 | glutathione S-transferase 1-like |  | evm.model.ctg8.219 | cytochrome P450 313a7 |
|  | evm.model.ctg28.20 | glutathione S-transferase D7 isoform X1 |  | evm.model.ctg1.91 | cytochrome P450 302a2 |
|  | evm.model.ctg244.6 | glutathione S-transferase zeta 1 |  | evm.model.ctg54.20 | cytochrome P450 12a3 |
|  | evm.model.ctg93.16 | glutathione S-transferase 1-1-like isoform X2 |  | evm.model.ctg142.36 | cytochrome P450 313a3 |
|  | evm.model.ctg315.16 | glutathione S-transferase 1-like |  | evm.model.ctg370.2 | cytochrome P450 313a2 |
|  | evm.model.ctg3.499 | glutathione S-transferase theta-1-like |  | evm.model.ctg142.35 | cytochrome P450 313a1 |
|  | evm.model.ctg19.10 | glutathione S-transferase 1-like |  | evm.model.ctg35.172 | cytochrome P450 28a6 |
|  | evm.model.ctg66.18 | glutathione S-transferase 1-like |  | evm.model.ctg106.12 | cytochrome P450 6a22 |
|  | evm.model.ctg282.33 | glutathione S-transferase D1-like |  | evm.model.ctg35.170 | cytochrome P450 28a7 |
|  | evm.model.ctg35.180 | glutathione S-transferase theta-1-like |  | evm.model.ctg138.37 | cytochrome P450 4d3 |
|  | evm.model.ctg3.250 | glutathione S-transferase-like |  |  |  |
