# Supplementary material for: A chromosome-level genome assembly provides insights into the environmental adaptability and outbreaks of Chlorops oryzae
Source: Commun Biol. 2022 Aug 26;5:881. doi: 10.1038/s42003-022-03850-7 (PMC9418232; doi:10.1038/s42003-022-03850-7)
Supplement: Supplementary file 6 — Supplementary Data 4 [file 42003_2022_3850_MOESM6_ESM.docx]

**Supplementary Data 4. Primers used in this study.**

| Purpose | Genes | Forward primers (5’-3’) | Reverse primers (5’-3’) |
| --- | --- | --- | --- |
| qPCR | *HSP83* | GCGACCAGTGACGATACACA | GCTTTCGCTTGAGCTGTTGT |
|  | *HSP70* | AGCTGCCAAATGCTGTTCCTA | TTGAAAATCTGCGCGGCAC |
|  | *HSP68* | ATCTTCGGAGTCAAGCAGGC | CCTTTTCAGCGGTCGTGTTG |
|  | *HSP67B2* | ATTCATTGCTCTTTCGGCAGC | ACCAGATTCTCCGACCAGTG |
|  | *HSP27* | TCGTCGCATGTGCCAACTAT | TCGTGCTTACCCTCAACGAC |
|  | *HSP23* | GTTTGGCCGACGATTTGGAC | AGGCTGCATGGCACGATTTA |
|  | *SOD* | TAGTGCGGCCAATGATGCTA | GCAACCAAGGTATTTCGAGGG |
|  | *GST* | GCCGGAGCATGAGAAAAGTTC | ATACCAAGATGGCCTGTGCC |
|  | *POD* | TCGGAGGCTCTCTGGAATCA | GTCTCCGGTTCGTGTTCGAT |
|  | *RPS15* | TCCGGCAAGATCGTCGTCAA | CTGACGCGAAGGCAGCAAAT |
|  | *RP49* | TCTTGCGAACGTAGACGACC | CGGTGAGATTGCCCATGGTGT |
|  | Vg | GCACAAAATGCGAGACGGAG | CCAACCGGAGACGAGGAAAA |
|  | Met | TGCCAATCCGACCTGTATGG | CCTTCGTCGTCATTTGTGCG |
|  | Tai | GCCAATCAGGGCTTTCAACG | TTGTTGTTGGCGAAACGCAC |
|  | Kr-h1 | GGACTTGCACGAAGCCAATG | TTCTCGTTGCTGAGAAGCGT |
|  | USP | ATCGAGCGGAATTGTCATGC | TGTTCGTCCAAGCAAGCGTA |
|  | InR | ACAGGGCTCATTCGGTATGG | CGGTTGCGTTCTCATTGACC |
|  | PI3K | CCAACACAAACGACCGGAAC | TGAGTTCGATGCAGCACCTT |
|  | FOXO | CGTGAGGGTCTATCGCCAAA | CTTCAGTCGGAACGCCATCT |
|  | TOR | TGGACGGTTAACTCACCACTG | ATGCAGCAGTACGCGTAAGA |
| RNAi | Vg | GGATCCTAATACGACTCACTATAGGGTTTCGTGTGCTCATAGTCGC | GGATCCTAATACGACTCACTATAGGCCCAGAAGTGCTCCTTCTAAGTT |
|  | Met | GGATCCTAATACGACTCACTATAGGCTAGCTTGCCGCACAATGG | GGATCCTAATACGACTCACTATAGGCATTTGCTTCATGGCAGTCGT |
|  | Tai | GGATCCTAATACGACTCACTATAGGTTTCCGACGATCAGCCCTTT | GGATCCTAATACGACTCACTATAGGAGTTGTTGTTGGCGAAACGC |
|  | Kr-h1 | GGATCCTAATACGACTCACTATAGGAGGACTTGCACGAAGCCAA | GGATCCTAATACGACTCACTATAGGAGCACAACTTCCGCTTACTCTA |
|  | USP | GGATCCTAATACGACTCACTATAGGTTGCTGATAGCCAATGTTGCG | GGATCCTAATACGACTCACTATAGGCTAGCATGAGGTGTTCTAATGGCT |
|  | InR | GGATCCTAATACGACTCACTATAGGGCTAAAGACGCGGTTGAAGAA | GGATCCTAATACGACTCACTATAGGACCGGCCTCGTTAAGGAAAT |
|  | PI3K | GGATCCTAATACGACTCACTATAGGTGAACGACGTCTACCCGAGAT | GGATCCTAATACGACTCACTATAGGGTGTGCCCTTCTGTTGTCAAG |
|  | FOXO | GGATCCTAATACGACTCACTATAGGTGCACAGTGGTGGTTTCCAA | GGATCCTAATACGACTCACTATAGGACCATCAGGCTAACGGCTTC |
|  | TOR | GGATCCTAATACGACTCACTATAGGGACTTTCGAATGGACGAAACCG | GGATCCTAATACGACTCACTATAGGTGAATTGTCATGCAGCAGTACG |

Notes: T7 RNA polymerase promoter sequence is underlined.
