# Supplementary material for: A chromosome-level genome assembly provides insights into the environmental adaptability and outbreaks of Chlorops oryzae
Source: Commun Biol. 2022 Aug 26;5:881. doi: 10.1038/s42003-022-03850-7 (PMC9418232; doi:10.1038/s42003-022-03850-7)
Supplement: Supplementary file 7 — Reporting summary [file 42003_2022_3850_MOESM7_ESM.pdf]

nature portfolio

Corresponding author(s): Lin Qiu and Youzhi Li

Last updated by author(s): Jun 10, 2022

Reporting Summary

Nature Portfolio wishes to improve the reproducibility of the work that we publish. This form provides structure for consistency and transparency in reporting. For further information on Nature Portfolio policies, see our Editorial Policies and the Editorial Policy Checklist.

Statistics

For all statistical analyses, confirm that the following items are present in the figure legend, table legend, main text, or Methods section.

n/a	Confirmed	
☐	☒	The exact sample size (*n*) for each experimental group/condition, given as a discrete number and unit of measurement
☐	☒	A statement on whether measurements were taken from distinct samples or whether the same sample was measured repeatedly
☐	☒	The statistical test(s) used AND whether they are one- or two-sided<br>*Only common tests should be described solely by name; describe more complex techniques in the Methods section.*
☐	☒	A description of all covariates tested
☐	☒	A description of any assumptions or corrections, such as tests of normality and adjustment for multiple comparisons
☐	☒	A full description of the statistical parameters including central tendency (e.g. means) or other basic estimates (e.g. regression coefficient) AND variation (e.g. standard deviation) or associated estimates of uncertainty (e.g. confidence intervals)
☐	☒	For null hypothesis testing, the test statistic (e.g. *F*, *t*, *r*) with confidence intervals, effect sizes, degrees of freedom and *P* value noted<br>*Give P values as exact values whenever suitable.*
☐	☒	For Bayesian analysis, information on the choice of priors and Markov chain Monte Carlo settings
☐	☒	For hierarchical and complex designs, identification of the appropriate level for tests and full reporting of outcomes
☐	☒	Estimates of effect sizes (e.g. Cohen's *d*, Pearson's *r*), indicating how they were calculated

Our web collection on statistics for biologists contains articles on many of the points above.

Software and code

Policy information about availability of computer code

Data collection	we used the follwing software to collect the data in this manuscript: Canu, in-built High Quality Region Finder, BLASR (v5.1), Arrow (v2.2.1) , BWA (v0.7.9a) , Pilon (v1.22), HiC-Pro (v2.7.8), RepBase , RepeatMasker , RepeatProteinMask, ab initio, RepeatModeler, RepeatMasker, PASA, Genewise, Augustus, SNAP, GeneMark, EVidenceModeler, OrthoMCL
Data analysis	GraphPad Prism 8 and OmicShare tools were used for data analysis

For manuscripts utilizing custom algorithms or software that are central to the research but not yet described in published literature, software must be made available to editors and reviewers. We strongly encourage code deposition in a community repository (e.g. GitHub). See the Nature Portfolio guidelines for submitting code & software for further information.

Data

Policy information about availability of data

All manuscripts must include a data availability statement. This statement should provide the following information, where applicable:

- Accession codes, unique identifiers, or web links for publicly available datasets
- A description of any restrictions on data availability
- For clinical datasets or third party data, please ensure that the statement adheres to our policy

All the data mentioned above are available in our manuscript

Human research participants

Policy information about studies involving human research participants and Sex and Gender in Research.

Reporting on sex and gender	The material used in our manuscript is insect, this information has not been collected
Population characteristics	The material used in our manuscript is insect, this information has not been collected
Recruitment	The material used in our manuscript is insect, this information has not been collected
Ethics oversight	The material used in our manuscript is insect, this information has not been collected

Note that full information on the approval of the study protocol must also be provided in the manuscript.

Field-specific reporting

Please select the one below that is the best fit for your research. If you are not sure, read the appropriate sections before making your selection.

☒ Life sciences ☐ Behavioural & social sciences ☐ Ecological, evolutionary & environmental sciences

For a reference copy of the document with all sections, see nature.com/documents/nr-reporting-summary-flat.pdf

Life sciences study design

All studies must disclose on these points even when the disclosure is negative.

Sample size	Twenty Chlorops oryzae larvae were used for Illumina sequencing for transcriptome analysis, and 100 female adults were collected for Illumina, PacBio, and Hi-C sequencing for genome analysis.
Data exclusions	no data were excluded from the analyses
Replication	Tree replication were applied for transcriptome analysis, all attempts at replication were successful
Randomization	all sample were randomly allocated
Blinding	The investigators were blinded to group allocation during data collection and analysis

Reporting for specific materials, systems and methods

We require information from authors about some types of materials, experimental systems and methods used in many studies. Here, indicate whether each material, system or method listed is relevant to your study. If you are not sure if a list item applies to your research, read the appropriate section before selecting a response.

Materials & experimental systems

n/a	Involved in the study	
☒	☐	Antibodies
☒	☐	Eukaryotic cell lines
☒	☐	Palaeontology and archaeology
☐	☒	Animals and other organisms
☒	☐	Clinical data
☒	☐	Dual use research of concern

Methods

n/a	Involved in the study	
☒	☐	ChIP-seq
☒	☐	Flow cytometry
☒	☐	MRI-based neuroimaging

Animals and other research organisms

Policy information about studies involving animals; ARRIVE guidelines recommended for reporting animal research, and Sex and Gender in Research

Laboratory animals	The study did not involve laboratory animals
Wild animals	The organism used in this study is insect Chlorops oryzae (Diptera: Ephydridae), the study did not involved wild animals

Reporting on sex	Female adults were determined according to the size and shape of genitalia and then 100 females were used for genome sequencing
Field-collected samples	Chlorops oryzae larvae were collected in 2019 in Hanshou County, Hunan province, China, and reared on fresh rice stems in the laboratory. Larvae were kept at 24 ± 1 °C and > 80% relative humidity, under a photoperiod of 16:8 (L:D) h.
Ethics oversight	The organism used in this study is insect, no ethical approval or guidance was required

Note that full information on the approval of the study protocol must also be provided in the manuscript.
